# Non-perfectly matching small RNAs can induce stable and heritable epigenetic modifications and can be used as molecular markers to trace the origin and fate of silencing RNAs

**DOI:** 10.1093/nar/gkab023

**Published:** 2021-02-01

**Authors:** Yue Fei, Tünde Nyikó, Attila Molnar

**Affiliations:** University of Edinburgh, Institute of Molecular Plant Sciences, Max Born Crescent, Edinburgh EH9 3BF, UK; Agricultural Biotechnology Institute, Szent-Györgyi A. 4., Gödöllő 2100, Hungary; University of Edinburgh, Institute of Molecular Plant Sciences, Max Born Crescent, Edinburgh EH9 3BF, UK

## Abstract

Short non-coding RNA molecules (sRNAs) play a fundamental role in gene regulation and development in higher organisms. They act as molecular postcodes and guide AGO proteins to target nucleic acids. In plants, sRNA-targeted mRNAs are degraded, reducing gene expression. In contrast, sRNA-targeted DNA sequences undergo cytosine methylation referred to as RNA-directed DNA methylation (RdDM). Cytosine methylation can suppress transcription, thus sRNAs are potent regulators of gene expression. sRNA-mediated RdDM is involved in genome stability through transposon silencing, mobile signalling for epigenetic gene control and hybrid vigour. Since cytosine methylation can be passed on to subsequent generations, RdDM contributes to transgenerational inheritance of the epigenome. Using a novel approach, which can differentiate between primary (inducer) and secondary (amplified) sRNAs, we show that initiation of heritable RdDM does not require complete sequence complementarity between the sRNAs and their nuclear target sequences. sRNAs with up to four regularly interspaced mismatches are potent inducers of RdDM, however, the number and disruptive nature of nucleotide polymorphisms negatively correlate with their efficacy. Our findings contribute to understanding how sRNA can directly shape the epigenome and may be used in designing the next generation of RNA silencing constructs.

## INTRODUCTION

Short (21–24 nt) non-coding RNAs (sRNAs) play a fundamental role in gene regulation in eukaryotes. They are generated from the cleavage of partially or perfectly matched double-stranded RNA (dsRNA) by DICER or Dicer-like (DCL) nucleases and are subsequently loaded into ARGONAUTE (AGO) proteins. AGO proteins are guided by the sRNAs to nucleic acid targets by base-pair complementarity. Thus sRNAs act as molecular postcodes. If the target is mRNA, its cleavage, destabilization, or translational inhibition results in post-transcriptional gene silencing (PTGS). If the target is chromatin, the AGO–sRNA complex induces epigenetic change through cytosine methylation of DNA. This process is known as RNA-directed DNA methylation (RdDM). RdDM in nuclear promoter regions often result in transcriptional gene silencing (TGS) thus cytosine methylation plays an important role in regulating gene expression (1–3).

Canonical RdDM is mediated by 24 nt sRNAs that are generated via the RNA POLYMERASE IV (Pol IV)–RNA-DEPENDENT RNA POLYMERASE 2 (RDR2)–DICER-LIKE NUCLEASE 3 (DCL3) pathway (4) from repeat sequences including transposons (5) and transgenes (6), with the help of the CLASSY SNF2-related chromatin remodeler family (CLSY) involved in global Pol IV recruitment (7). The 24 nt sRNAs guide AGO effector proteins (AGO4, 6, 9) (8–10) to longer non-coding RNA transcripts produced by RNA POLYMERASE V (Pol V) (11,12) and accessory proteins including DNA methylation readers SUVH2 and SUVH9 (13), and chromatin-remodelling complex components such as RNA-DIRECTED DNA METHYLATION 1 (RDM1), DEFECTIVE IN MERISTEM SILENCING 3 (DMS3) and DEFECTIVE IN RNA-DIRECTED DNA METHYLATION 1 (DRD1) (14,15). Subsequent recruitment of DOMAIN REARRANGED METHYLTRANSFERASE 2 (DRM2) catalyses *de novo* DNA methylation at the target locus (16). Maintenance of DNA methylation on newly synthesised DNA involves DNA METHYLTRANSFERASE 1 (MET1) and CHROMOMETHYLASE 3 (CMT3) (17), which reproduce CG and CHG methylation, respectively.

In ‘non-canonical RdDM’, sRNAs can be alternatively generated from viral RNA or RNA POLYMERASE II (Pol II) transcripts via diverse RDR–DCL pathways and subsequently loaded onto AGO4, 6 and 9 to interact with Pol V RNAs (18).

The 24 nt sRNAs play an important role both in inter- and intra-genomic interactions including hybrid vigour (19,20) and genome imbalance in triploid endosperm of *Arabidopsis* seeds (21–23). Transposon-derived sRNAs have been involved in driving the evolution of gene expression in plants (24). Importantly, recent experiments demonstrated that 24 nt sRNAs are mobile in plants and can direct TGS in recipient tissues including meristems (25,26). Since meristems give rise to new organs including flowers, mobile 24 nt sRNAs can initiate epigenetic changes that may persist and yield heritable (trans-generational) phenotypes.

PTGS, which is mediated by 21–22 nt sRNAs, can operate with up to five mismatches between the sRNA and its target mRNA (27–29). In contrast, the complementarity requirement between 24 nt sRNAs and Pol V RNAs leading to AGO4-mediated DRM2 recruitment has not been systematically tested. Early work, preceding the discovery of sRNAs found that a mutated 35S promoter lacking CG/CNG methylation acceptor sites became susceptible to TGS when a transgenic plant harbouring this locus was crossed with a non-mutated 35S silencer line (30). This now suggests that 24 nt sRNAs produced by the silencer line were able to induce DNA methylation without complete complementarity to the target locus. However, the inducer and the target sequence shared four regions (60, 28, 33 and 47 nt) with 100% homology, which did not exclude the possibility that initiation of RdDM relies on perfectly matching sRNAs.

Here we show that initiation of trans-generational RdDM does not require 100% sequence complementarity between the sRNAs and their nuclear target sequences. In addition, we demonstrate that non-perfectly matching sRNAs can be used as tools to fine map the production and spread of sRNAs that are associated with many RNA silencing pathways.

## MATERIALS AND METHODS

### Plant material

*Nicotiana benthamiana* (line 16c), harbouring the Green Fluorescent Protein (*GFP*) transgene under the control of the Cauliflower Mosaic Virus (CaMV) 35S promoter was previously described (31). *Arabidopsis thaliana* carrying the *fwa-d* epimutation in the Columbia ecotype (Col *fwa-d*) was previously described (32). All plants were grown in Levington F2+S professional growth compost in a controlled growth chamber (SANYO/Panasonic) at 22°C with 16-h light and 8-h dark periods. *Arabidopsis* seeds were stratified for 48 h in darkness at 4°C prior to planting. Leaf samples for RNA and DNA analysis were collected at different time points as described in the figure legends.

### Flowering time assessment

Flowering time was measured by counting the number of primary rosette leaves at the time of bolting. According to the number of leaves, plants were sorted into three groups as described previously (33). Early, intermediate and late flowering refers to plants with ≤16, 17–22 and ≥23 leaves at the time of bolting, respectively. Approximately, 48 individual plants were analysed from each line.

### Construction of tobacco rattle virus (TRV)-based virus induced gene silencing (VIGS) vectors

To construct TRV vectors for *GFP* silencing, 20 μM of 120 nt oligonucleotides matching the CaMV 35S promoter (-208 to -89, (34)), the *GFP5* coding sequence (+364 to +483, (31)) and their derivatives harbouring single-nucleotide substitutions (SNSs) at regular intervals were mixed with the corresponding reverse complement oligonucleotides in 1× NEB Buffer 3 (New England Biolabs, NEB) in a 25 μl reaction volume. To anneal the oligonucleotides, the mixtures were incubated at 98°C for 5 min and then slowly cooled down (-0.3°C/sec) to room temperature. 1 μl of dsDNA was phosphorylated with T4 Polynucleotide Kinase (1 U/μl, NEB) in 1× T4 DNA ligase buffer (NEB) and subsequently ligated into the SmaI site of Tobacco Rattle Virus (TRV) vector pTRV2 (35) to generate vectors: TRV-35S, TRV-35S-1M_A, TRV-35S-1M_B, TRV-35S-2M, TRV-35S-4M, TRV-35S-1M_TV, TRV-35S-2M_TV, TRV-GFP, TRV-GFP-2M and TRV-GFP-2M_TV.

For *FWA* silencing, TRV-FWA-B was obtained from Dr Donna M. Bond as described previously (note that TRV-FWA-B corresponds to TRV:FWAtr) (33). FWA-B is a 544-nt fragment of the *FWA* promoter (At4g25530), which contains two tandem repeats. A shorter version of FWA-B, referred to as FWA-Bs that harbours a single short and long repeat (239 nt) was PCR amplified and subsequently ligated into the SmaI site of pTRV2 to generate TRV-FWA-Bs. The FWA-Bs derivatives harbouring SNSs at regular intervals were generated by assembling the corresponding oligonucleotides (top, middle and bottom) in 1× Phusion HF Buffer, 20 nM of each oligonucleotides (top, middle and bottom), 250 μM dNTPs, 1 unit of Phusion DNA Polymerase in 50 ul final volumes. The mixtures were incubated in a thermal cycler at 98°C 90 sec > (98°C 30 sec > 55°C 30 sec > 72°C 30 sec) × 5, then 0.2 uM of corresponding forward and reverse oligonucleotides were added into each reaction to amplify the DNA at 98°C 90 sec > (98°C 30 sec > 55°C 30 sec > 72°C 30 sec) × 30 > 72°C 10 min. The PCR products were gel purified, phosphorylated and cloned into the SmaI site of pTRV2, to create TRV-FWA-Bs-1M_A, TRV-FWA-Bs-1M_B and TRV-FWA-Bs-2M, respectively. Oligonucleotides used for generating the viral vectors are listed in Supplementary Table S2.

### Viral inoculations

To generate infectious viruses, *Agrobacterium tumefaciens* (strain GV3101:pMP90 + pSOUP) was first transformed with the binary vectors containing TRV RNA1 (pTRV1), TRV RNA2 (pTRV2) and recombinant pTRV2s (see above). *A. tumefaciens* carrying TRV1 or any form of pTRV2 were mixed at a 1:1 ratio and then co-infiltrated into the leaves of 4-week-old *N. benthamiana* plants as described previously (36). To infect *Arabidopsis thaliana*, systemic leaves of infiltrated *N. benthamiana* plants were collected at 7 days post infection (dpi) and ground in 1 mM sodium phosphate buffer (pH 7.0) to obtain viral sap. Three rosette leaves of 4-week-old *Arabidopsis* plants were rub-inoculated with 10 μl of viral sap, using aluminium oxide as an abrasive.

### Imaging of GFP fluorescence

GFP expression was monitored under UV light using a handheld mercury UV lamp (UVP, B-100AP Lamp 100 W 365 nm). Photographs were taken using a Canon G16 camera. Camera exposure settings were *f*/3.2, ranging from 3 to 6 sec, depending on the intensity of GFP fluorescence and distance from the plant.

### Quantitative RT-PCR

Total nucleic acid (TNA) was purified from leaf tissue by phenol–chloroform extraction as described (37). For cDNA synthesis, 3 μg of TNA was treated with Turbo DNase (Ambion) according to the manufacturer's instruction. cDNA was then synthesised from 1 μg of RNA using random hexamers and Superscript II (Life Technologies). Quantitative RT-PCR analysis was carried out with SYBR Green I Master Mix on a LightCycler®480 instrument (Roche) using gene-specific oligonucleotides (Supplementary Table S2). *N. benthamiana ACTIN* and *A. thaliana EF1a* were used for normalization, respectively. Three technical replicates were performed for each biological replicate. The following cycling conditions were used for all reactions: 95°C 5 min > (95°C 10 sec > 60°C 10 sec > 72°C 15 sec) × 45.

### DNA methylation analysis using Bisulfite sequencing

DNA samples were prepared from ∼100 mg of leaf tissue using GenElute™ Plant Genomic DNA Miniprep Kit (SIGMA-ALDRICH). Approximately 400 ng of DNA was treated with bisulfite reagent according to the EZ DNA Methylation-Gold Kit (Zymo Research). Approximately 50 ng of DNA was amplified by One Taq Hot Start DNA polymerase (NEB) using gene-specific oligonucleotides (Supplementary Table S2). The PCR products were cloned into the pGEM-T Easy vector (Promega). *Escherichia coli* (DH5alpha strain) cells were transformed with the recombinant DNA and then spread on LB-agar plates supplemented with 50 μg/ml carbenicillin. Eight to 16 clones from each sample were sequenced by BigDye 3.1 (Thermo Fisher Scientific) according to manufacturer instructions. Bisulfite-converted sequences were aligned to the corresponding sequences using Clustal Omega (http://www.ebi.ac.uk/Tools/msa/clustalo/). DNA methylation patterns were analysed by the CyMate software (38).

### Small RNA cloning and bioinformatics analysis

Small RNA libraries were prepared from 1 μg of TNA using the Illumina TruSeq Small RNA Library Prep Kit and sequenced on the Illumina HiSeq 2500 platform with 50-base single end reads at Edinburgh Genomics (Edinburgh, UK). Sequence analysis was performed using the Geneious software (version 11.1.4, http://www.geneious.com). Briefly, after removing the adaptor sequences, size-selected reads between 21 and 24 nt were mapped either to recombinant TRV RNA2 or to the transgenic *N. benthamiana* 16c T-DNA+partialTn5393 locus (GenBank Accession No. KY464890) and its derivatives, where target sequences were modified according to the mutations that were introduced into the recombinant TRV trigger (TRV-35S-2M and TRV-GFP-2M). Only perfectly matching small RNAs were included in our analyses. The small RNA sequencing data are available in the ArrayExpress database under accession number E-MTAB-8342.

## RESULTS

### Developing a TRV-based VIGS system to study the impact of mismatched sRNAs on RdDM initiation at the 35S transgene locus

**Figure 1.**
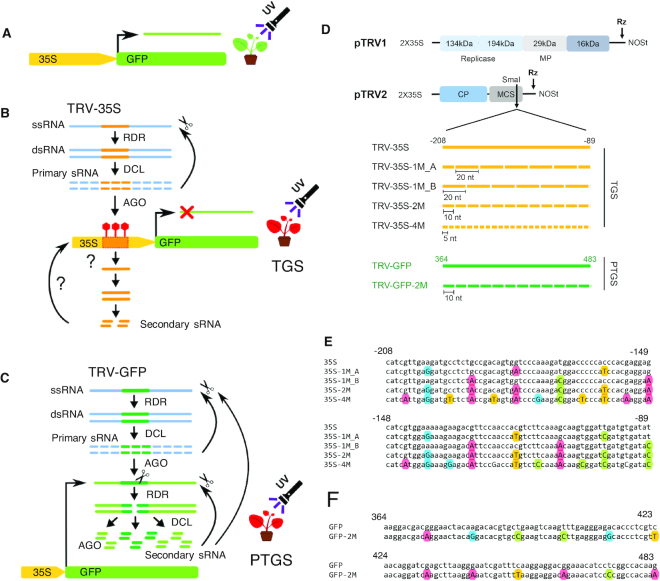
TRV-based VIGS system used for studying the impact of mismatched small RNAs on silencing of the *GFP* transgene. (**A**) Schematic diagram of the CaMV 35S promoter driven *GFP* transgene in *N. benthamiana* 16c plant. 16c plants show green fluorescence under UV light. (**B**) Schematic diagram of virus induced TGS. Reproduction of RNA viruses, such as TRV results in the accumulation of double-stranded replication intermediates, which are processed into primary sRNAs by antiviral DICER-like (DCL) nucleases. sRNAs are then associated with and guide ARGONAUTE (AGO) proteins to nucleic acid targets by base-pair complementarity. If the target is chromatin, sRNAs can induce the methylation of cytosine residues. This process is referred to as RdDM. Consequently, inoculation of 16c plants with a recombinant TRV carrying the 35S promoter sequence (TRV-35S) can bring about RdDM, which results in TGS of the *GFP* reporter gene. *GFP* silenced 16c plants display red fluorescence under UV-light due the autofluorescence of chlorophyl in lack of *GFP* expression. It is not known whether RdDM of 35S is associated with the production of secondary (target-generated) sRNAs. sRNA-induced DNA methylation is indicated as red lollipop. RDR, RNA-dependent RNA polymerase (**C**) Schematic diagram of virus induced PTGS. Virus-derived primary sRNAs can also be loaded into AGO complexes to destroy target RNAs with complementary sequences. It is known as antiviral PTGS, which involves AGO-induced cleavage, destabilization, or translational inhibition. Infecting 16c plants with a recombinant TRV harbouring the *GFP* coding sequence (TRV-GFP) can result in PTGS of both TRV and *GFP* mRNAs. Intriguingly this process is associated with the generation of secondary (*GFP*-specific) sRNA. sRNA-induced cleavage is indicated as scissors. (**D**) Schematic diagram of the TRV VIGS vectors pTRV1 and pTRV2. A 120 nt fragment of the CaMV 35S promoter (-208 to -89 relatives to the transcription start site, yellow lines) or a 120 nt fragment of the *GFP* coding sequence (+364 to +483, green lines) was cloned into pTRV2 to induce TGS and PTGS, respectively. Single nucleotide substitutions (SNS; white boxes) were introduced into the 120 nt fragments at regular intervals of 20, 10 or 5 nucleotides, which produced sRNAs with one, two or four mismatches, respectively. SNS were introduced from position 10 in TRV-35S-1M_A and from position 20 in TRV-35S-1M_B. pTRV1 was used along with pTRV2 to generate functional TRV particles. Rz, self-cleaving ribozyme; MCS, multiple cloning sites; CP, coat protein; MP, movement protein; NOSt, NOS terminator. (**E**) Sequence alignment of the 120 nt fragment from CaMV 35S and its derivatives from (A). Substituted A, C, G, T nucleotides are highlighted with red, green, blue and yellow coloured circles, respectively. (**F**) Sequence alignment of the 120 nt fragment from *GFP5* and its derivative from (A).

To set up a system where we could examine gene silencing, we used *GFP* driven constitutively by the CaMV 35S (35S) promoter as a reporter gene in transgenic *N. benthamiana* (16c, (31) Figure 1A). A 120 bp segment of either the 35S promoter or *GFP* coding sequence were targeted to induce TGS and PTGS, respectively. To bypass the variance in transgene-derived sRNAs due to transgene rearrangements and positional effects, we delivered the sRNA precursors via recombinant RNA viruses (Figure 1B and C). Our rationale was that the nuclear and the antiviral silencing pathways overlap and RNA virus-derived sRNAs can induce RdDM and subsequently TGS with high efficiency (39,40). Indeed, a recombinant TRV carrying the full 35S promoter sequence, and a Cucumber Mosaic Virus harbouring a shorter 120 bp segment of 35S, had been shown to trigger sequence-specific DNA methylation and heritable transcriptional gene silencing (34,39,41).

To systematically investigate the specificity and activity of RdDM-inducing sRNAs, we designed sRNAs with mismatches to their target sequence. As a template for sRNA production, we used a recombinant TRV containing a 120 bp segment of the 35S promoter (TRV-35S). We then created a series of variants carrying single-nucleotide substitutions (SNSs) at every 20, 10 or 5 nucleotides (nt) within this segment. The sRNAs produced from these vector variants would have at least one (TRV-35S-1M), two (TRV-35S-2M) or four (TRV-35S-4M) mismatches to the 35S target segment, respectively (Figure 1D and E). To test the effect of the relative position of the SNSs, we produced two versions of TRV-35S-1M where the SNSs were shifted by 10 nt relative to each other (TRV-35S-1M_A, TRV-35S-1M_B) (Figure 1E). In the first set of experiments, SNSs were designed to introduce G↔A and C↔T substitutions. Thus sRNAs that are randomly generated from viral dsRNA precursors could pair with their target nucleic acid via non-Watson-Crick base paring referred to as G:U wobble (GUW). GUW can result in thermostability in RNA-RNA interactions and has been shown to lead to miRNA-target RNA recognition (42,43). However, a G-U mismatch in the seed region may potentially interfere with target binding (44), and the efficiency of miRNA-mediated repression is reduced as more GUWs are introduced (28,43). In plants, mismatches in miRNA in positions 9−11 strongly impair AGO1-mediated cleavage while they have little effect when located in the 3' end (45–48). Interestingly, siRNA-guided AGO4-mediated cleavage of Pol V transcripts has also been implicated in RdDM (49). Although siRNA target recognition and efficiency of silencing might be governed by different principles than that of miRNAs, our SNS design strategy ensures that polymorphic nucleotides are introduced in every virus-derived sRNA with having the least possible impact on sRNA-mediated cleavage.

### Non-perfectly matching sRNAs can induce stable RdDM at 35S without secondary sRNA accumulation

**Figure 2.**
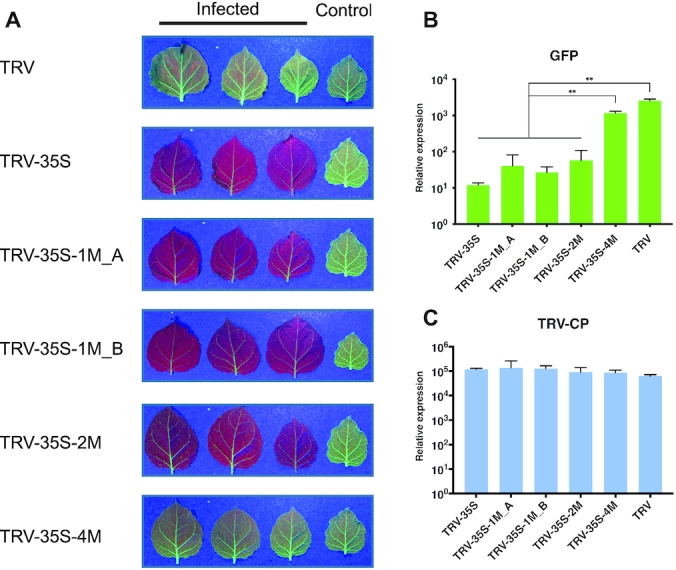
Non-perfectly matching virus-derived small RNAs can induce strong transcriptional gene silencing. (**A**) Systemic leaves of *N. benthamiana* 16c plants infected with recombinant TRV as indicated. Leaves were collected from independent plants. An uninfected 16c leaf is shown as a control (right). Leaves were photographed at 21 dpi under UV light. (**B** and **C**) Analysis of *GFP* expression and TRV accumulation in recombinant TRV-infected plants by qRT-PCR. RNA was extracted from the systemically infected leaves at 21 dpi. Error bars show the standard error of the mean (SEM) of three independent biological replicates. Asterisks indicate significant differences (Student's *t*-test, *P* < 0.01). No spontaneous *GFP* silencing was observed neither in 16c plants nor in plants infected with wild type TRV.

We infected 16c plants (31) with wild type and recombinant TRVs and monitored *GFP* expression under UV light after infection. We found that the unmodified 35S segment was sufficient to induce strong *GFP* silencing in TRV-35S infected plants (seen in Figure 2A as red chlorophyll fluorescence in the absence of *GFP* expression), as reported for CMV (34). Intriguingly, SNSs in the sRNAs did not prevent silencing. Plants infected with TRV-35S-1M_A, TRV-35S-1M_B and TRV-35S-2M showed similar levels of *GFP* silencing as plants inoculated with the non-mutated TRV-35S vector, while TRV-35S-4M caused a low level of *GFP* silencing. The relative positions of the SNSs in TRV-35S-1M_A and TRV-35S-1M_B had no effect on silencing (Figure 2A). To further investigate the impact of virus infection on *GFP* expression, we isolated RNA from leaves infected systemically 3 weeks post inoculation and assessed *GFP* and viral RNA accumulation by quantitative reverse-transcription PCR (qRT-PCR). In agreement with the phenotypic data, *GFP* silencing was associated with reduced *GFP* mRNA levels. We detected at least 50 times less *GFP* mRNA in TRV-35S, TRV-35S-1M and in TRV-35S-2M infected tissues compared to wild type TRV (Figure 2B). The lower level of silencing induced by TRV-35S-4M (Figure 2B) was not due to reduced infection or stability of the virus (Supplementary Figure S1) because TRV-35S-4M samples contained similar level of viral RNA as tissues infected with other recombinant TRVs (Figure 2C). From these experiments we concluded that 35S promoter-targeting sRNAs do not require 100% sequence complementarity to induce TGS.

**Figure 3.**
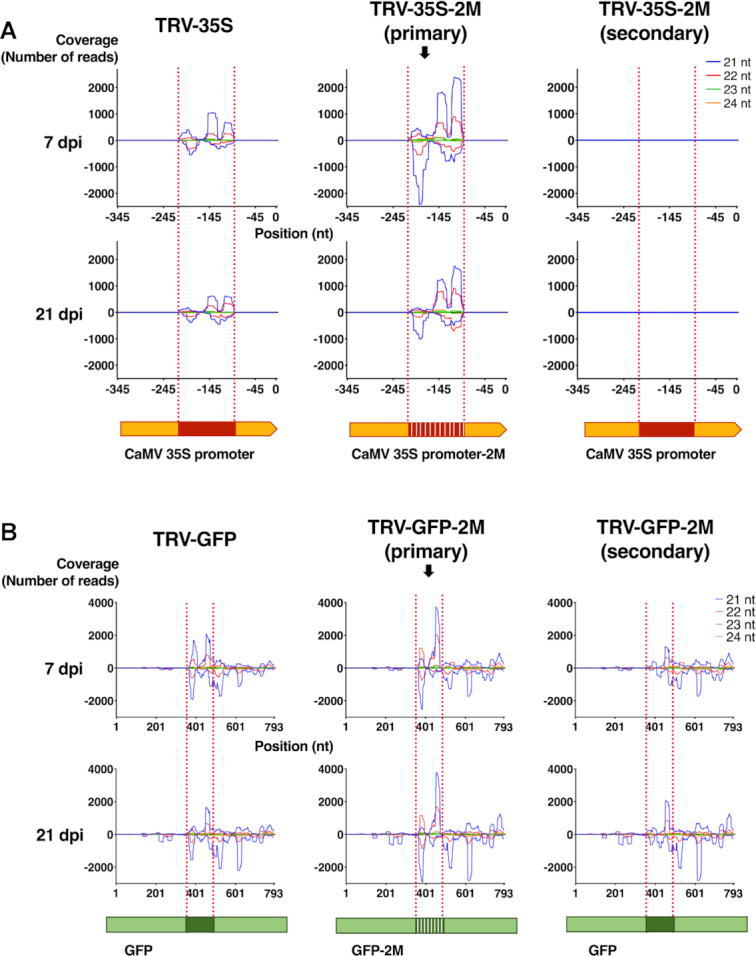
Virus-induced TGS is not associated with the accumulation of secondary small RNAs. (**A**) Small RNA analysis of *N. benthamiana* 16c plants infected with TGS-inducing TRV-35S and TRV-35S-2M at 7 dpi and 21 dpi. sRNA reads were aligned to the 35S promoter or to 35S promoter-2M harbouring the corresponding SNSs. sRNAs from TRV-35S-2M infected plants were separated according to SNS content to yield primary (containing SNSs) and secondary sRNAs (lacking SNSs). The numbers of sRNAs mapping at each position of the plus strand are shown as positive values, to the minus as negative values, for 21, 22, 23 and 24 nt sRNAs separately. The target sequence is highlighted by dotted lines. (**B**) Small RNA analysis of *N. benthamiana* 16c plants infected with PTGS-inducing viruses TRV-GFP and TRV-GFP-2M at 7 dpi and 21 dpi. sRNA reads were aligned to the *GFP* coding sequence and to its variant GFP-2M containing the corresponding SNSs. sRNAs from TRV-GFP-2M infected plants were separated into primary and secondary sRNAs according to SNS content. Labelling as in (A).

To gain further insight into viral sRNA production and sRNA-promoter interaction, we sequenced sRNAs from virus-infected tissues at an early (7 dpi) and late time point (21 dpi). TRV-35S-2M was chosen for this experiment because it contains SNSs at 10 nt intervals and so generates sRNAs with two distinct mismatches to the 35S target sequence. This allows virus-derived primary sRNAs to be distinguished with more certainty from secondary sRNAs. The latter are produced from the endogenous target sequence in an RDR-dependent manner as part of the signal amplification process that is associated with the plant RNA silencing pathways. Hence, secondary RNAs will not contain SNSs, even if they are induced by primary sRNAs that do contain them. We used the unmodified TRV-35S as a control. To assess the quality of our sRNA libraries, we first aligned sRNA sequences to the TRV genome (RNA2, Supplementary Figure S2A). As expected, TRV infection was associated with the accumulation of virus-specific sRNAs. In accordance with the literature (50) we found that sRNAs were not distributed evenly along the viral genome, likely due to a combination of differences in processing of the viral RNA, sRNA stability and cloning bias. Nevertheless, the sRNA profile was nearly identical in all samples (Supplementary Figure S2A and B). Moreover, the number of TRV-matching sRNAs and the relative abundance of sRNAs derived from the 35S segment were similar in each sRNA library (Supplementary Table S1) indicating that the recombinant TRVs were potent inducers of the antiviral RNA silencing machinery regardless of the SNS content (35S or 35S-2M). To separate the virus-derived primary sRNAs from the target-generated secondary sRNAs, we separated the reads according to SNS content by aligning them to the target 35S locus (Figure 3A). In the control TRV-35S infected 16c plants, where primary and secondary sRNAs could not be distinguished, we detected high levels of 35S-specific sRNAs in all sizes indicating that multiple Dicers could act on the sRNA precursor (Figure 3A). Interestingly, these sRNAs aligned exclusively to the 120 nt segment of 35S that was used as a silencing inducer in TRV-35S and did not extend into the rest of the 35S sequence present in the target (Figure 3A, left panels). The same sRNA distribution was observed for primary sRNAs from TRV-35S-2M infected plants (Figure 3A, middle panels). In addition, only a handful of secondary sRNAs were identified in TRV-35S-2M infected plants (Figure 3A, right panels, Supplementary Figure S3). Together, these data suggest that there was no or very limited transitivity (spreading of sRNAs beyond the target site) at the 35S locus even at 21 dpi, due to the lack of secondary sRNA production.

To exclude the possibility that mismatched sRNAs were unable to trigger secondary sRNA production and transitive RNA silencing, we generated another set of recombinant TRVs to induce silencing of the coding region of the *GFP* reporter gene via PTGS (Figure 1C, D and F). We used a 120 bp segment of the *GFP* gene and a variant containing SNSs at 10 nucleotide intervals to create TRV-GFP and TRV-GFP-2M, respectively. Infection of 16c plants using these vectors was expected to result in the activation of RNA silencing and the generation of *GFP*-specific primary sRNAs, which *in trans* can guide the sequence-specific degradation of *GFP* mRNAs and induce secondary sRNA production (51). Indeed, 16c plants infected with TRV-GFP and TRV-GFP-2M resulted in strong *GFP* silencing (Supplementary Figure S4). No phenotypic difference was observed between the TRV-GFP and TRV-GFP-2M infection, suggesting that SNSs in sRNAs do not inhibit the initiation or progression of PTGS and hence secondary sRNA generation. We sequenced sRNAs from the TRV-GFP and TRV-GFP-2M infected 16c plants at 7 and 21 dpi, aligned reads to the *GFP* coding region and separated primary and secondary sRNAs as before (Figure 3B). As in the TRV-35S experiments, we found that the recombinant TRVs activated antiviral RNA silencing, which resulted in the accumulation of 21–24 nt sRNAs specific to TRV (Supplementary Figure S2B) or the *GFP* insert (Figure 3B, Supplementary Table S1). However, in contrast to TGS via promoter targeting (Figure 3A), secondary sRNA production was associated with and not restricted to the targeted *GFP* region in TRV-GFP and TRV-GFP-2M infected tissues (Figure 3B). This indicates that both TRV-GFP and TRV-GFP-2M were capable of inducing transitivity and the production of secondary sRNAs. Hence, SNSs are unlikely to have caused the lack of transitivity we observed in our TGS experiments. As a control, we also sequenced sRNAs from wild type TRV-infected *N. benthamiana* 16c plants and aligned them both to the 35S promoter and the *GFP* coding region (Supplementary Figure S5). We detected a single sRNA that matched the 35S promoter and tens of sRNAs that mapped evenly along the *GFP* coding region, likely as a result of transgene transcript degradation. This data indicates that the 120 nt segments in the recombinant TRV are necessary and sufficient to trigger sRNA-mediated silencing. From the above experiments we conclude that non-perfectly matching sRNAs can induce gene silencing even if they target promoter sequences. However, unlike the sRNAs involved in mRNA degradation (via PTGS), promoter-associated sRNAs (inducing TGS) appear to be exempt from further amplification.

**Figure 4.**
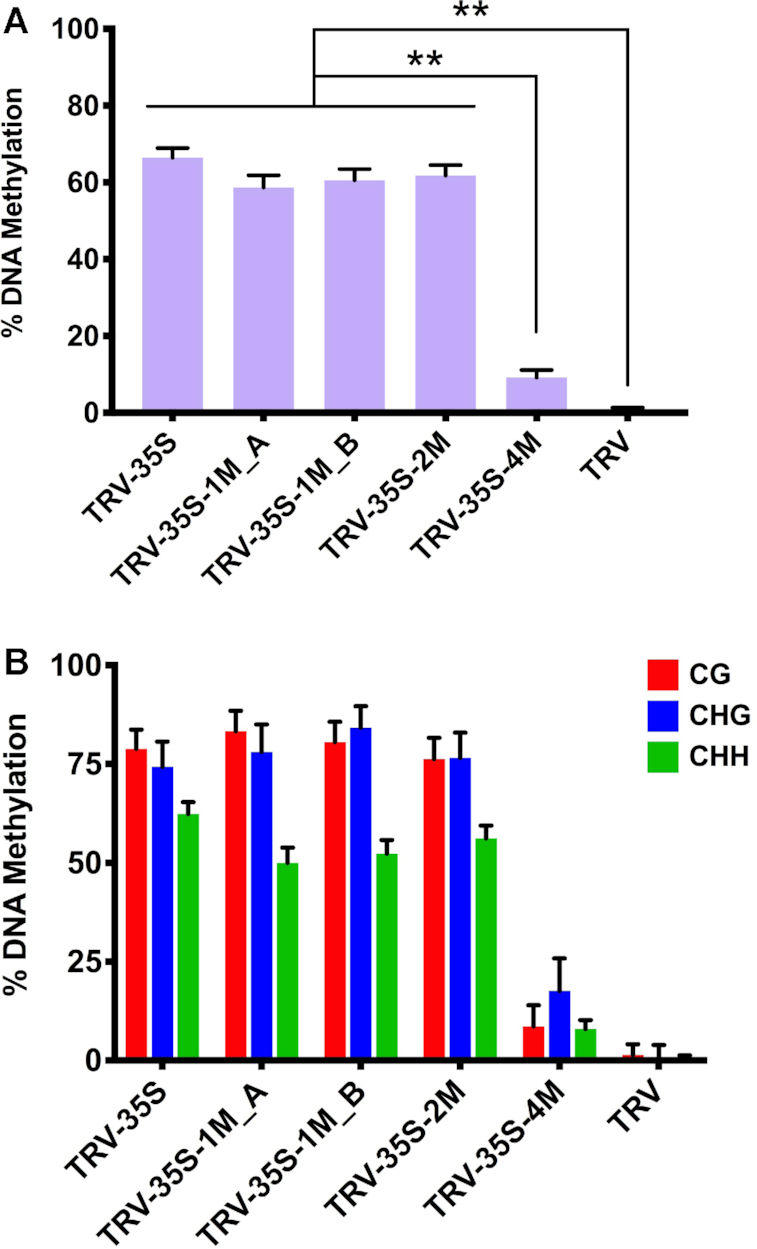
Non-perfectly matching small RNAs can efficiently induce DNA methylation in virus-infected plants. (**A**) Analysis of DNA methylation at the target CaMV 35S promoter (from -208 to -89) by bisulfite sequencing in TRV-infected *N. benthamiana* 16c plants. DNA was extracted from the systemically infected leaves at 21 dpi. The histogram shows the percentage of total methylated cytosine. Asterisks indicate significant differences (Student's *t*-test, *P* < 0.01). (**B**) Summary of bisulfite sequencing analysis. Red, blue and green bars indicate the percentage of methylated cytosine residues at CG, CHG and CHH sites, repetitively. Data presented in (A) and (B) were obtained from three independent biological replicates. Raw data are available in Supplementary Figure S6. Error bars represent a confidence interval with 95% confidence limits (Wilson score interval; see details in Supplementary Figure S6B).

To investigate the effect of mismatches on sRNA-mediated DNA methylation, we isolated DNA from tissues infected with TRV, TRV-35S, TRV-35S-1M_A and B, TRV-35S-2M and TRV-35S-4M (Figure 2A) and used bisulfite sequencing to assess the level and context of cytosine methylation at the 35S target sequence. As expected, wild type TRV did not induce DNA methylation (Figure 4, Supplementary Figure S6). In contrast, infection with TRV-35S was associated with a high level of cytosine methylation, further supporting that the 120 nt 35S promoter sequence was a potent activator of RdDM. TRV-35S-1M, TRV-35S-2M and TRV-35S-4M, which produced mismatched sRNAs, were also able to direct DNA methylation of the nuclear target promoter (Figure 4, Supplementary Figure S6). Together with the phenotypic data (Figure 2A), this shows that sRNAs with one or two mismatches can induce strong silencing via RdDM. Interestingly, sRNAs with four mismatches that greatly impaired the targeting ability of AGO were still capable of initiating low levels of RdDM (Figure 4B).

### Mismatched sRNA-induced RdDM of 35S is heritable to the next generation

**Figure 5.**
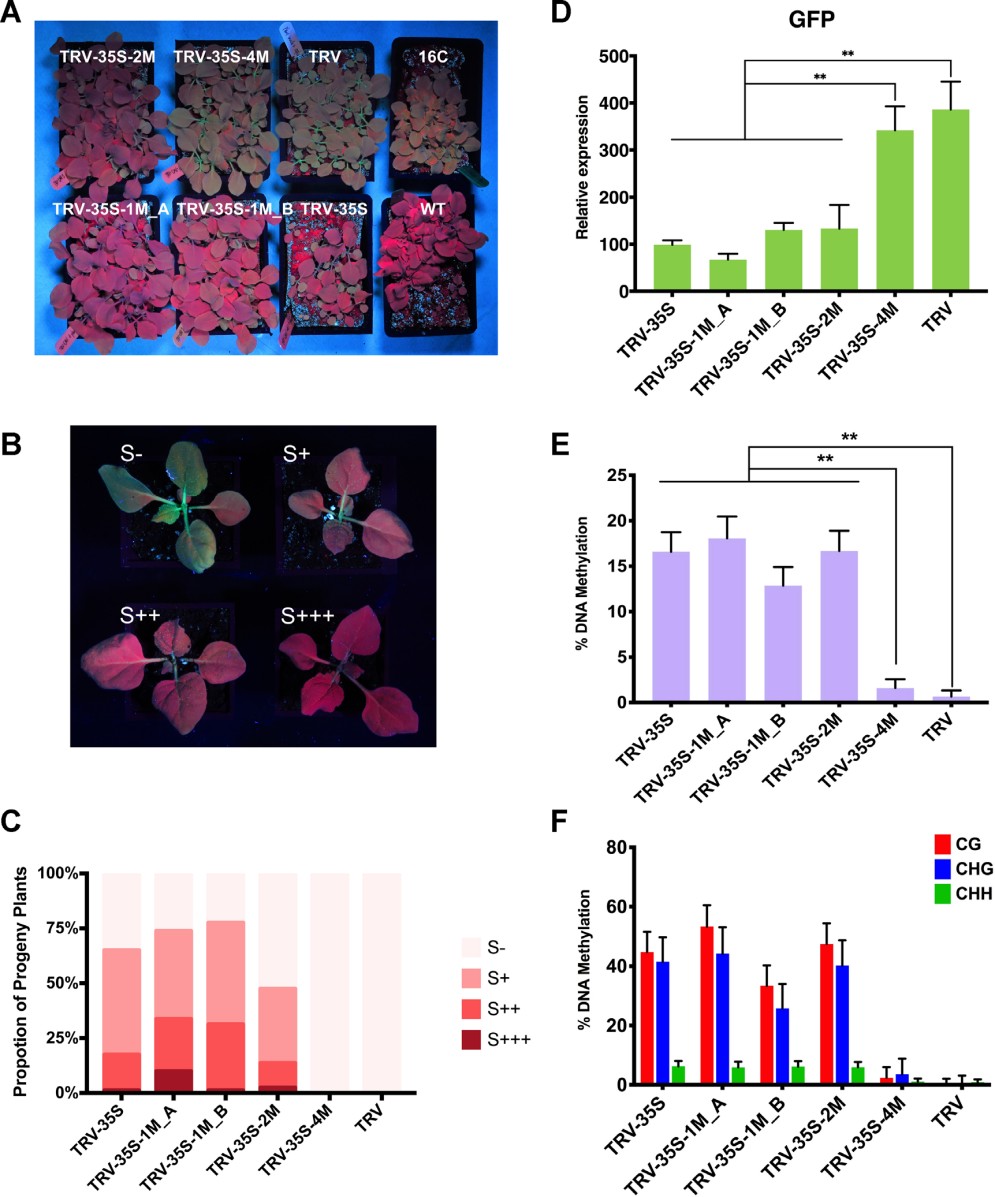
Non-perfectly matching small RNAs can induce transgenerational epigenetic silencing of the 35S:GFP reporter gene. (**A**) Progeny of recombinant-TRV-infected *N. benthamiana* 16c plants. Wild type *N. benthamiana* and uninfected 16c plants are shown as controls. Plants were photographed under UV light at 20 days after germination. (**B**) Representative plants displaying different degree of *GFP* silencing in the progeny of recombinant-TRV-infected *N. benthamiana* 16c plants: S+++, full *GFP* silencing; S++, *GFP* silencing in leaves and in one pair of petioles; S+, *GFP* silencing only in leaves; S-, no visible sign of *GFP* silencing. Plants were photographed under UV light at 22 days after germination. (**C**) Proportion of plants in each silencing category. Forty individual plants were analyzed from each line in two independent biological replicates. Bars represent average values. (**D**) Analysis of *GFP* expression in the progeny of recombinant TRV-infected plants by qRT-PCR. RNA was extracted from 21-day-old plants. Error bars show the standard error of the mean (SEM) of three independent biological replicates. Asterisks indicate significant differences (Student's *t*-test, *P* < 0.01). (**E**) Analysis of DNA methylation at the CaMV 35S promoter (from -208 to -89) by bisulfite sequencing in the progeny of recombinant-TRV-infected *N. benthamiana* 16c plants. DNA was extracted from 21-day-old plants. The histogram shows the percentage of total methylated cytosine. Asterisks indicate significant differences (Student's *t*-test, *P* < 0.01). (**F**) Summary of bisulfite sequencing analysis. Red, blue and green bars indicate the percentage of methylated cytosine residues at CG, CHG and CHH sites, repetitively. Results presented in (E) and (F) were obtained from three independent biological replicates. Raw data are available in Supplementary Figure S6. Error bars represent a 95% interval (Wilson score interval; see details in Supplementary Figure S6B).

To test the effect of mismatched sRNAs on transgenerational epigenetic gene silencing, we analysed the virus-free progeny of 16c plants infected with wild type and recombinant TRVs. By screening the young seedling of the progeny, we found that silencing of the reporter gene with TRV-35S, TRV-35S-1M and TRV-35S-2M was passed to the next generation (Figure 5A), suggesting that non-perfectly matching sRNAs were able to induce heritable TGS. However, some plants exhibited *GFP* de-repression (expression). To investigate the penetrance of *GFP* silencing, we sorted the progeny of recombinant TRV-infected plants into four categories based on the level and spatiotemporal domain of *GFP* repression: S+++ plants showed full *GFP* silencing, S++ plants displayed *GFP* silencing in leaves and in one pair of petioles, S+ plants revealed *GFP* silencing only in leaves, while S- plants demonstrated no *GFP* silencing (Figure 5B). We found that the S+++ and S++ plants maintained *GFP* silencing throughout their development, whereas the S+ group reverted to the green fluorescence phenotype. Around one fifth of the TRV-35S progeny fell into the strong *GFP* silencing categories (S+++ and S++) (Figure 5C), which is in line with previous observations (39). Similarly, 14%–34% of the progeny of TRV-35S-2M and TRV-35S-1M infected plants displayed stable *GFP* repression, which indicates that the reduction in CHH methylation accompanying the TRV-35S-1M and -2M infections had no effect on the inheritance of *GFP* silencing. Interestingly, sRNAs with one mismatch (TRV-35S-1M_A and TRV-35S-1M_B) slightly increased the frequency of heritable TGS. Detailed analysis of *GFP* expression (Figure 5D) and promoter DNA methylation (Figure 5E–F, Supplementary Figure S6A) revealed a strong inverse correlation in the progeny and reduced *GPF* expression was always accompanied by high levels of symmetric cytosine methylation (CG and CHG) regardless of the number of mismatches (one or two) in the sRNA inducer of TGS.

### Non-perfectly matching sRNAs can induce RdDM at the *FWA* endogene

**Figure 6.**
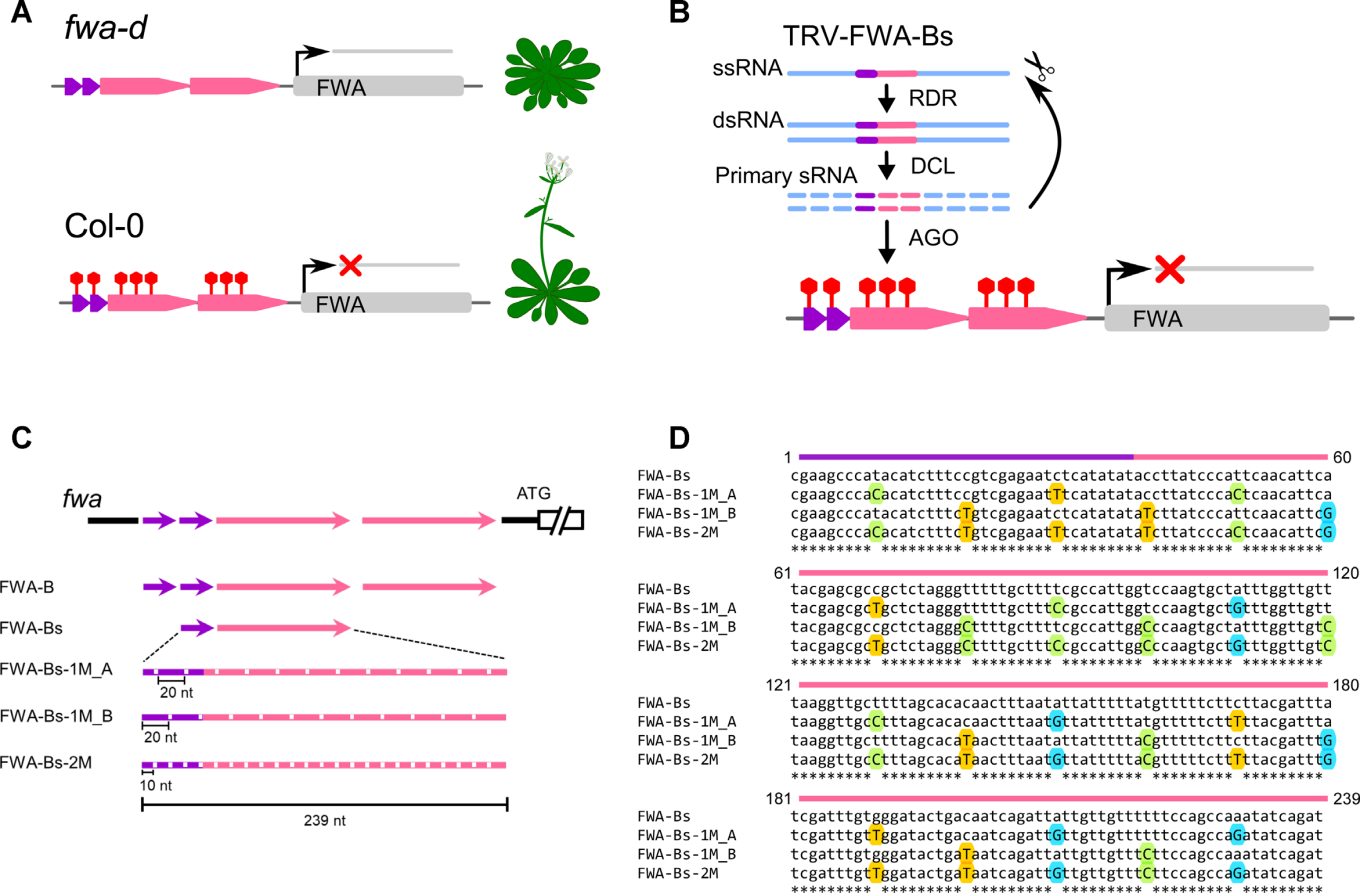
TRV-based VIGS system used for transcriptional silencing of the *FWA* endogene. (**A**) Schematic diagram of the *FWA* locus in *Arabidopsis thaliana*. FWA is a suppressor of flowering time. In wild-type *A. thaliana* (Col-0), the direct tandem repeats in the *FWA* promoter are hyper-methylated which results in transcriptional gene silencing of *FWA*. Repression of *FWA* is associated with early flowering phenotype. In the *fwa-d* epigenetic mutant, DNA methylation at direct tandem repeats is reduced and consequently *FWA* is expressed. Ectopic expression of *FWA* results in late flowering phenotype. (**B**) Experimental design to induce TGS of *FWA* by infecting *fwa-d* with a recombinant TRV harbouring a short and a long repeat sequence referred to as Bs (TRV-FWA-Bs). (**C**) Schematic diagram of the tandem repeats in *FWA* promoter (At4g25530) and the repeat-derived sequences used for VIGS. The short (38 nt) and long (198 nt) repeats are indicated as purple and pink arrows, respectively. A 239 nt fragment of the *FWA* promoter harbouring a single short and long repeat sequence referred to as FWA-Bs was cloned into pTRV2 to induce TGS. SNSs were introduced at regular intervals of 20 and 10 nucleotides to generate sRNAs with one or two mismatches, respectively. SNSs were inserted from position 10 in FWA-Bs-1M_A and from position 20 in FWA-Bs-1M_B. The full-length tandem repeat, referred to as FWA-B (32) or FWAtr (33) was used as a positive control. pTRV1 was utilised along with pTRV2 to generate functional TRV particles. (**D**) Sequence alignment of FWA-Bs and its derivatives from (A). Substituted A, C, G, T nucleotides are highlighted with red, green, blue and yellow coloured circles, respectively. The short and long repeat sequences are indicated as purple and pink arrows, respectively.

**Figure 7.**
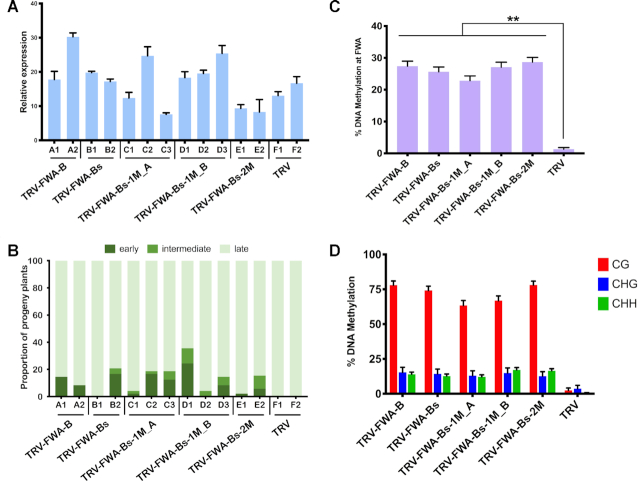
Non-perfectly matching sRNAs targeting the *FWA* promoter can induce early flowering in the progeny of virus-infected *Arabidopsis* plants. (**A**) Analysis of virus accumulation in recombinant-TRV-infected Col-0 *fwa-d* plants by qRT-PCR. RNA was extracted from leaf tissues at 28 dpi. Error bars show the standard error of the mean (SEM) of three technical replicates. Individual plants are labelled with a combination of capital letters and numbers. (**B**) Proportion of early- (dark green), intermediate- (green), and late- (light green) flowering plants in the progeny of recombinant-TRV-infected Col-0 *fwa-d* plants (A). (**C**) Analysis of DNA methylation at the *FWA* promoter (full length *FWA* tandem repeats) by bisulfite sequencing in the progeny of recombinant-TRV-infected Col-0 *fwa-d* plants. DNA was extracted and analysed from three individual plants from each selected line 40 days after germination. The histogram shows the percentage of total methylated cytosine residues. Asterisks indicate significant differences (Student's *t*-test, *P* < 0.01). (**D**) Summary of bisulfite sequencing analysis. Red, blue and green bars indicate the percentage of methylated cytosine at CG, CHG and CHH sites, repetitively. Raw data are available in Supplementary Figure S8. Error bars represent a confidence interval with 95% confidence limits (Wilson score interval; see details in Supplementary Figure S8C).

To investigate the capacity of mismatched sRNAs for initiating TGS of an endogenous gene, we set up a VIGS experiment to induce *de novo* DNA methylation at the promoter of the *FLOWERING WAGENINGEN* (*FWA*) gene. FWA is a homeobox-leucine-zipper protein involved in controlling flowering time as a suppressor. In wild-type *Arabidopsis thaliana* (Col-0), *FWA* is naturally silenced through hyper-methylation of direct tandem repeats in the *FWA* promoter. In the *fwa-d* epigenetic mutant, the loss of DNA methylation at direct tandem repeats is associated with ectopic expression of *FWA*, which results in a late flowering phenotype (32) (Figure 6A). Infection of *fwa-d* with a recombinant TRV harbouring full length *FWA* tandem repeats (TRV-FWAtr) can induce progressive silencing of *FWA* likely in the germ line (33). Consequently, the progeny of infected plants are hyper-methylated at the TRV-FWAtr target site (also known as fragment B; (32)), and display an early-flowering phenotype. Fragment B consists of two short (38 nt) and two long (198 nt) tandem repeats. To reduce complexity in our experimental design, we cloned a short and a long repeat sequence referred to as B short (Bs, 239 nt) into TRV (TRV-FWA-Bs, Figure 6B and C). We then generated a series of variants carrying SNS at every 20 and 10 nucleotides within Bs following the nucleotide replacement rule described above. The sRNAs produced from these viral vectors would have at least one (TRV-FWA-Bs-1M) or two (TRV-FWA-Bs-2M) mismatches to the *FWA* target segment, respectively (Figure 6D). We also produced two versions of TRV-FWA-1M where the SNSs were shifted by 10 nt relative to each other (TRV-FWA-1M_A, TRV-FWA-1M_B) (Figure 6C and D). We omitted any recombinant viral constructs that would produce sRNAs with four mismatches since SNS at every 5 nucleotides greatly reduced the efficacy of RdDM on transgenes (Figure 4). We infected sixteen *fwa-d* plants with wild type and recombinant TRVs including TRV-FWA-B (TRV-FWAtr, (33)) as a control. Since a positive correlation has been observed between the level of TRV-FWAtr infection and the proportion of early flowering progeny (33), we first assessed the virus RNA accumulation by qRT-PCR in order to select the most infected plants for subsequent analyses (Figure 7A). We then monitored the flowering time of around 48 progeny plants (V_1_) from each selected line (Figure 7B, Supplementary Figure S7) by counting the number of rosette leaves at the time of bolting. In accordance with the literature we found that, unlike wild type TRV, TRV-FWAtr (TRV-FWA-B) infection resulted in accelerated flowering time in V_1_ as up to 17% of progeny flowered early. A similar number of early flowering plants was detected in the progeny of a TRV-FWA-Bs infected line (B2), which indicates that a single copy of the *FWA* tandem repeat was sufficient to induce trans-generational silencing. More importantly, the frequency of early flowering phenotype in the progeny of TRV-FWA-Bs-1M and TRV-FWA-Bs-2M infected plants was comparable to that of *fwa-d* inoculated with the non-mutated TRV-FWA-B or TRV-FWA-Bs (Figure 7 and Supplementary Figure S7). This data clearly shows that SNSs in the sRNAs did not prevent the epigenetic silencing of the endogenous *FWA* locus.

To examine the link between the early flowering phenotype and *de novo* DNA methylation of *FWA*, we isolated DNA from three individual plants in each early flowering group (Figure 7B, Supplementary Figure S7) and performed bisulfite sequencing. The early-flowering time in the V_1_ progeny of infected plants was associated with changes in DNA methylation at the TRV-FWA target sites (Figure 7C, Supplementary Figure S8). Hyper-methylation was established in all C sequence contexts, mostly at CG residues (Figure 7D). Interestingly, DNA methylation was also detected at the second, non-targeted long tandem repeat, which shares 95.43% homology with the *FWA*-derived repeat sequence used for producing the sRNAs (Supplementary Figure S8A and S9).

### Investigation of the impact of mismatch types on the efficacy of RdDM

**Figure 8.**
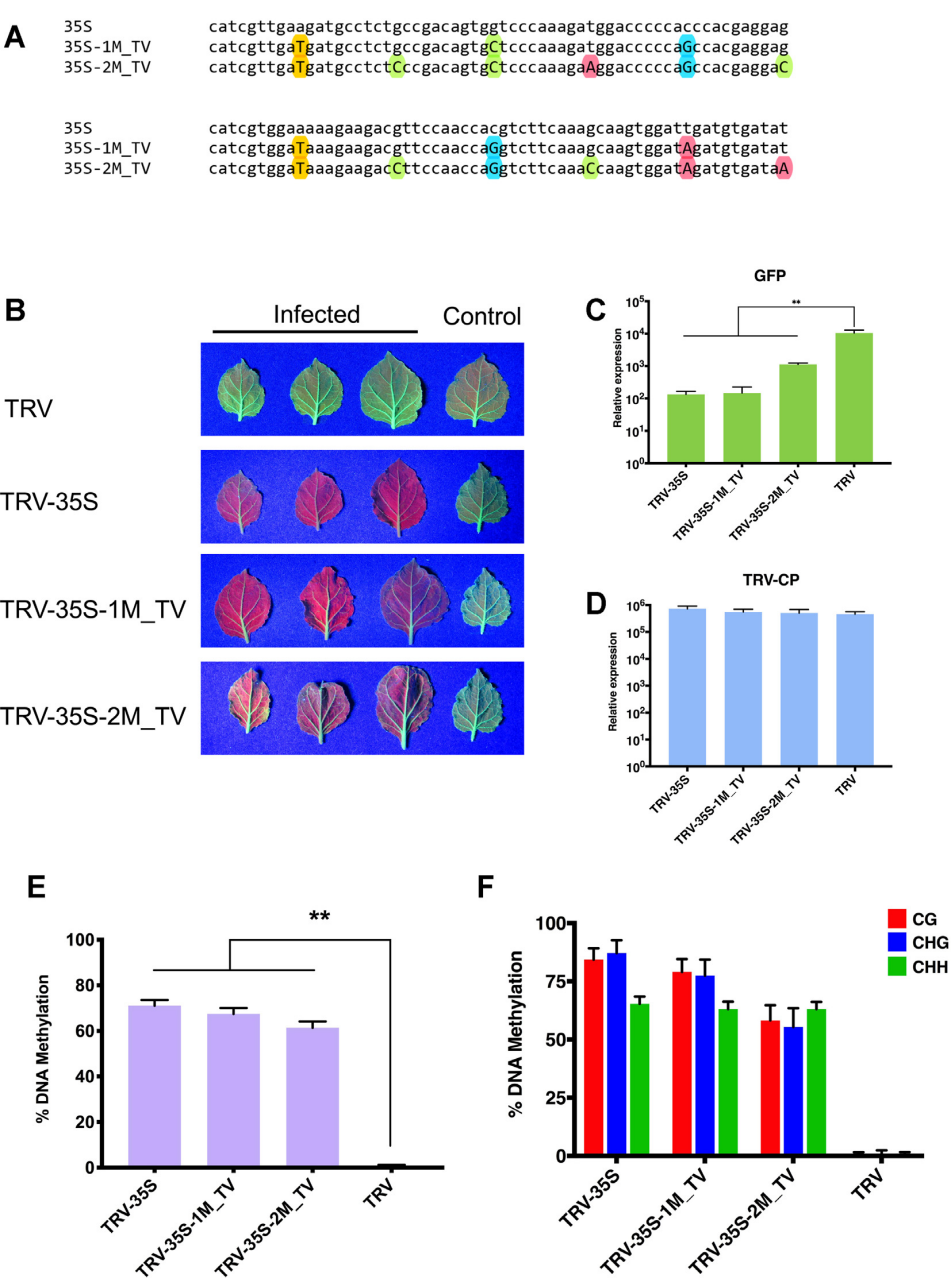
Small RNAs with transversal mismatches can also induce strong TGS. (**A**) Sequence alignment of the 120 nt fragment from CaMV 35S (from -208 to -89) and its derivatives. Substituted A, C, G, T nucleotides are highlighted with red, green, blue and yellow coloured circles, respectively. (**B**) Systemic leaves of *N. benthamiana* 16c plants infected with recombinant TRV as indicated. Leaves were collected from independent plants. An uninfected 16c leaf is shown as a control (right). Leaves were photographed at 21 dpi under UV light. (**C** and **D**) Analysis of *GFP* expression and TRV accumulation in recombinant TRV-infected plants by qRT-PCR. RNA was extracted from the systemically infected leaves at 21 dpi. Error bars show the standard error of the mean (SEM) of three independent biological replicates. Asterisks indicate significant differences (Student's *t*-test, *P* < 0.01). (**E**) Analysis of DNA methylation at the target CaMV 35S promoter (from -208 to -89) by bisulfite sequencing in TRV-infected *N. benthamiana* 16c plants. DNA was extracted from the systemically infected leaves at 21 dpi. The histogram shows the percentage of total methylated cytosine. Asterisks indicate significant differences (Student's *t*-test, *P* < 0.01). (**F**) Summary of bisulfite sequencing analysis. Red, blue and green bars indicate the percentage of methylated cytosine residues at CG, CHG and CHH sites, repetitively. Results presented in (E) and (F) were obtained from three independent biological replicates. Raw data are available in Supplementary Figure S10. Error bars represent a 95% interval (Wilson score interval; see details in Supplementary Figure S10B).

Finally, we tested the effect of non-GUW mismatches on the initiation and efficacy of TGS. To this end, we introduced transversal (TV) substitutions (A↔T and G↔C) into the 35S promoter segment at every 20 and 10 nt (35S-1M_TV and 35S-2M_TV, Figure 8A) and then we infected 16c plants with the corresponding TRV vectors (TRV-35S-1M_TV and TRV-35S-2M_TV). We found that both recombinant TRVs were able to induce strong silencing of the *GFP* reporter gene (Figure 8B–D), which were associated with high level of cytosine methylation at all sequence context at the targeted promoter DNA (Figure 8E, F and Supplementary Figure S10). From this experiment we conclude that sRNAs with up to two TV mismatches can induce strong silencing via RdDM. Interestingly, sRNAs harbouring two regularly interspaced TV SNS were less efficient silencers (TRV-35S-2M_TV, Figure 8C, F and Supplementary Figure S10) than the same sRNAs with two GUW mutations (TRV-35S-2M, Figure 2B, 4B and Supplementary Figure S6). This is in line with the more disruptive nature of TV mismatch than that of GUW in nucleic acid hybridization. However, we did not find any correlation between the GUW/TV mutations and the methylation level of corresponding cytosine residues (Supplementary Figure S11).

Overall, these experiments provide direct evidence that transgenerational RdDM does not require 100% sequence complementarity between the sRNAs and the target DNA sequence.

## DISCUSSION

### RdDM at the 35S locus

We demonstrate that the initiation step of RdDM can tolerate mismatches between the inducing sRNAs and the nuclear target sequence. Although there is still some debate whether sRNAs can directly target DNA (52), the current model (49) supported by experimental evidence suggests that sRNAs guide AGO proteins to nascent scaffold transcripts, recruiting DRM2, which methylates the previously unmodified cytosine residues in any sequence context (CG, CHG or CHH). The scaffold RNA can be transcribed either by the plant-specific Pol V (11,12,53) or less frequently by RNA polymerase II (Pol II) (54). Since Pol V recruitment requires DNA methylation (15) and both the 35S locus (Figure 4, 8 and Supplementary Figure S6 and S10) and the *fwa-d* promoter (Figure 7, Supplementary Figure S8) lack cytosine methylation, it is unlikely that Pol V transcripts are involved in the initiation step of virus-induced RdDM. Instead, we propose that Pol II transcripts could be the primary RNA targets in RdDM. The observation of bidirectional Pol II transcription around promoters (55,56) is consistent with this hypothesis. Once DRM2 is recruited and the corresponding cytosine residues are methylated, CG and CHG methylation can be maintained throughout cell division by MET1 and CMT3, respectively, even without the sRNA trigger. Indeed, this methylation pattern could be detected in the virus-free progeny of plants infected with recombinant TRV vectors (Figure 5F, Supplementary Figure S6, Figure 7D, Supplementary Figure S8), or using CMV (34), and is consistent with previous finding that virus-induced DNA methylation depends on MET1 for its transmission to the progeny (39).

In canonical RdDM, cytosine methylation is recognized by RNA polymerase IV (Pol IV), which generates a short 20–40 nt transcript that is subsequently converted into dsRNA by RNA-dependent RNA polymerase 2 (RDR2). Processing of this dsRNA by DCL3 gives rise to 24 nt sRNA(s), which guide AGOs to Pol V transcripts (∼200 nt), acting as scaffolds for recruitment of DRM2. The resulting DNA methylation can be recognised by complexes that modify the associated histones of chromatin. Likewise, proteins identifying specific histone modifications can recruit cytosine methyltransferases (57) leading to amplification of repression. Thus Pol IV and Pol V transcripts can define the genomic boundaries (58) and limit the spreading (transitivity) of RdDM and heterochromatin. Intriguingly, we found that virus-induced RdDM was restricted to the targeted region of 35S (Figure 4, 5, 8 and Supplementary Figure S6, S10) and cytosine methylation occurred and was maintained without the production of secondary sRNAs (Figure 3A, middle and right panel) suggesting that the Pol IV-Pol V-mediated amplification cycle of RdDM is impaired at the 35S locus in vegetative cells. Alternatively, this locus may not recruit Pol IV or Pol V at all, or sRNAs might invade the DNA without transcription to recruit DRM2 supporting the alternative model of RdDM (52). Interestingly, secondary sRNA production has been observed in transgene induced TGS (59) suggesting that spreading of epigenetic silencing might be influenced by the target locus-specific factors or the origin of primary sRNAs.

TRV-induced RdDM is correlated with the accumulation of 21–24 nt sRNAs suggesting that all DCLs can act on the precursor of sRNAs (this work and (33)). Recent work showed that VIGS-RdDM was enhanced in *dcl2,4* double mutants lacking most 22 nt and some 21 nt sRNAs (33), suggesting that TRV-mediated epigenetic silencing requires DCL3-generated 24 nt sRNAs and the associated AGO4. However, AGO1-bound 21nt sRNAs have been implicated in transposon silencing (54), therefore we cannot exclude the possibility that 21–22 nt sRNAs has a role in the early establishment of RdDM.

### The impact of mismatched sRNAs

Similar to sRNA-mediated PTGS, there is an inverse correlation between the number of mismatches and the efficacy of sRNA-mediated epigenetic gene silencing. We found that two mismatches had little effect on RdDM. In contrast, sRNAs with four mismatches greatly impaired the targeting ability of AGO. However, our virus-based sRNA delivery system does not allow us to investigate the impact of the position of mismatches in the sRNA on RdDM due to random processing of the sRNA precursor by antiviral DCLs. Hence further work is required to identify the specific nucleases and chromatin modifying complexes associated with virus-induced RdDM, and to define the targeting rule and the dose-response relationship of RdDM-inducing sRNAs.

Allowing mismatches in sRNA-mediated RdDM can increase the targeting space of the associated AGOs and DRMs. This could, for example, provide a more flexible control for fast evolving transposons or mitigate the effect of template-mismatched nucleotides at the 3′ end of sRNAs that are attributed to RDR2 terminal transferase activity (4). Our work demonstrates flexibility in sRNA-induced transgenerational epigenetic gene modifications and opens new avenues to investigate the intimate interaction between invading molecules such as transposons and viruses and the epigenome. In addition, it warrants more careful design and application of novel dsRNA sprays in plant protection (60,61) to avoid ‘off-target’ effects. Processing of exogenously applied antifungal or antiviral dsRNA *in planta* might result in sRNAs with partial complementarity to promoter sequences, which could induce heritable RdDM and consequently influence the expression of genes controlling agronomic traits (62).

Introducing regularly interspaced SNSs into the silencing trigger molecule allowed us to differentiate between primary and target-derived secondary sRNAs, and consequently to monitor the fate of sRNAs in different RNA silencing pathways. Since single SNSs had no impact on the activity of sRNA in either PTGS or TGS, our approach may be employed to study RNA signal amplification processes and RNA-RNA interaction pathways in other eukaryotes.

## DATA AVAILABILITY

The small RNA sequencing data are available in the ArrayExpress database under accession number E-MTAB-8342.

## Supplementary Material

gkab023_Supplemental_File
