# Supplementary material for: Non-perfectly matching small RNAs can induce stable and heritable epigenetic modifications and can be used as molecular markers to trace the origin and fate of silencing RNAs
Source: Nucleic Acids Res. 2021 Feb 1;49(4):1900–13. doi: 10.1093/nar/gkab023 (PMC7913690; doi:10.1093/nar/gkab023)
Supplement: gkab023_Supplemental_File [file gkab023_supplemental_file.pdf]

Supplementary Figure S1

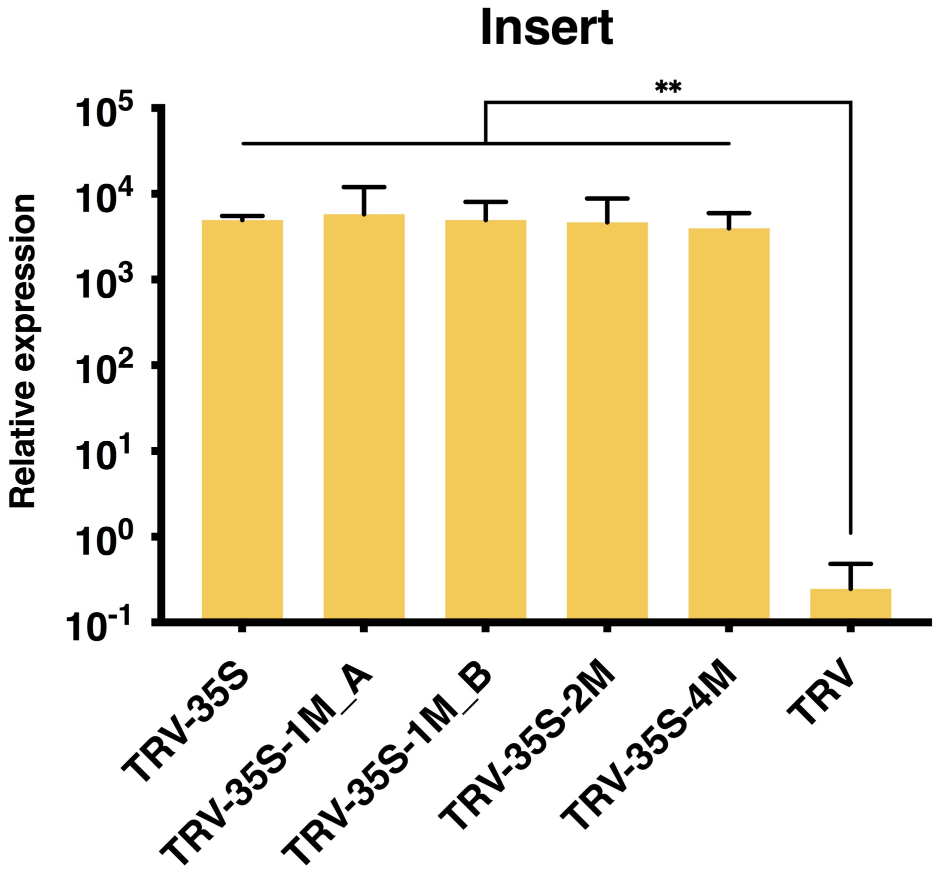

Supplementary Figure S1. Analysis of recombinant TRV in virus-infected *N. benthamiana* plants by qRT-PCR. The same RNA samples were used as in Figure 2. TRV RNA was amplified by insert-specific primers. Error bars show the standard error of the mean (SEM) of three independent biological replicates. Asterisks indicate significant differences (Student's T-test, $p < 0.01$).

Supplementary Figure S2

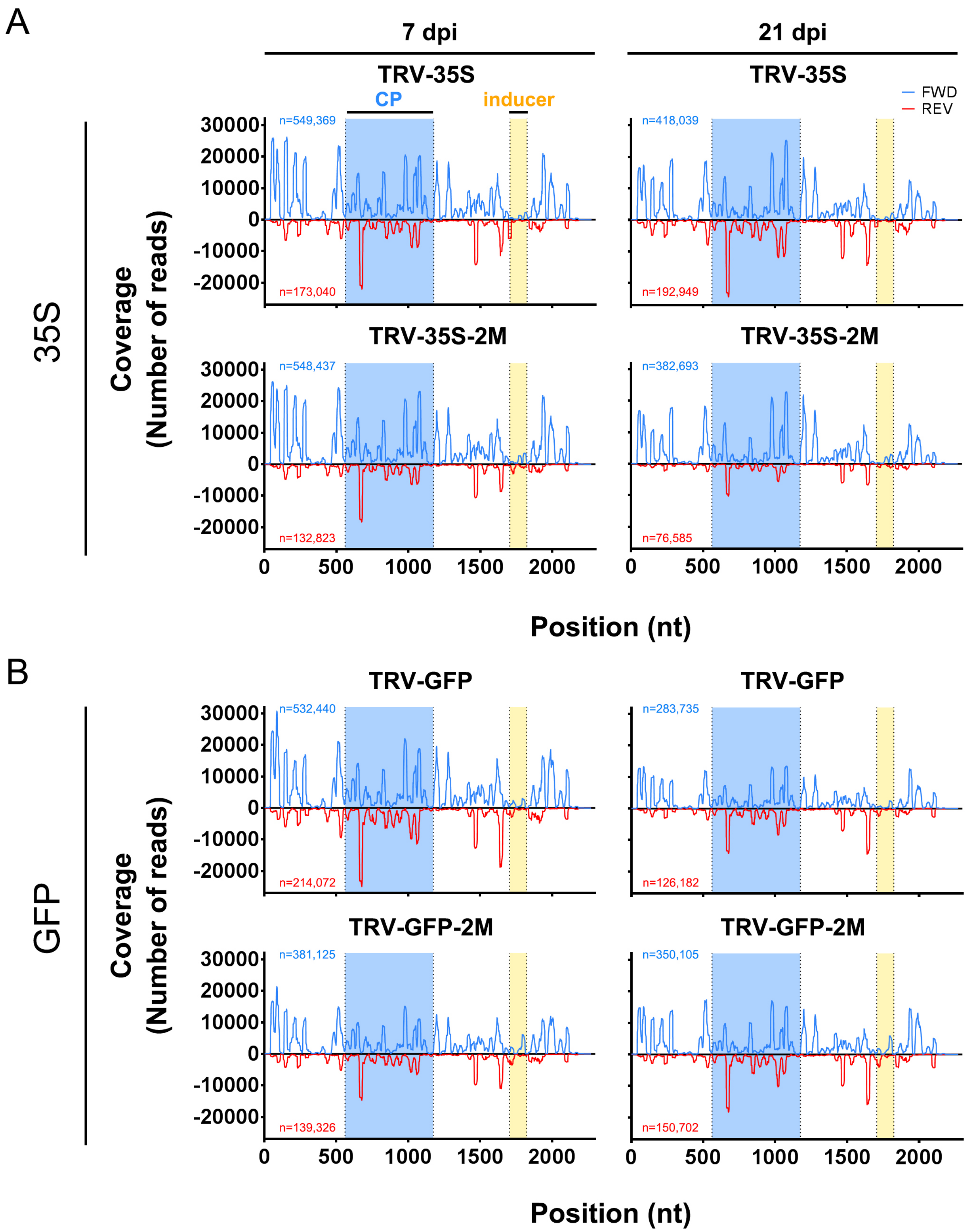

Supplementary Figure S2. Analysis of virus-derived small RNAs in recombinant-TRV-infected plants. Raw small RNA reads were aligned to the corresponding recombinant TRV RNA2 indicated. The positive or negative *y*-axis values show the number of small RNAs on either plus or minus strand as blue or red lines, respectively. The coat protein and the silencing-inducer sequences are highlighted as blue and yellow boxes, respectively. The total number of small RNAs matching the above selected regions of the viral genome is also indicated in the graph.

Supplementary Figure S3

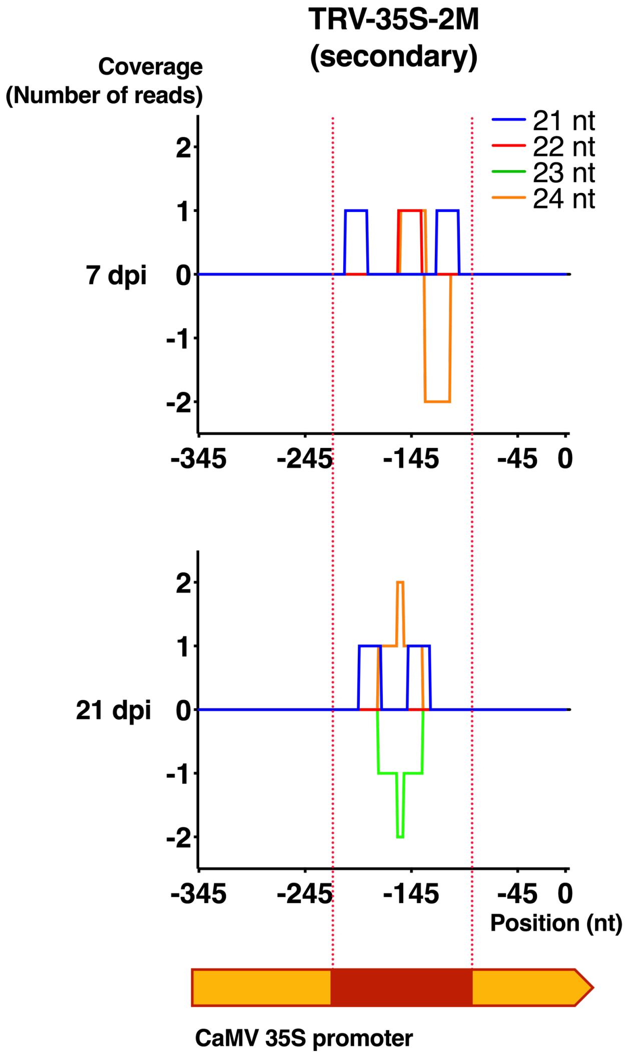

Supplementary Figure S3. Analysis of secondary small RNAs in TRV-35S-2M infected *N. benthamiana* 16c plants at 7 dpi and 21 dpi. High resolution map of small RNAs corresponding to Figure 3 top right panel.

Supplementary Figure S4

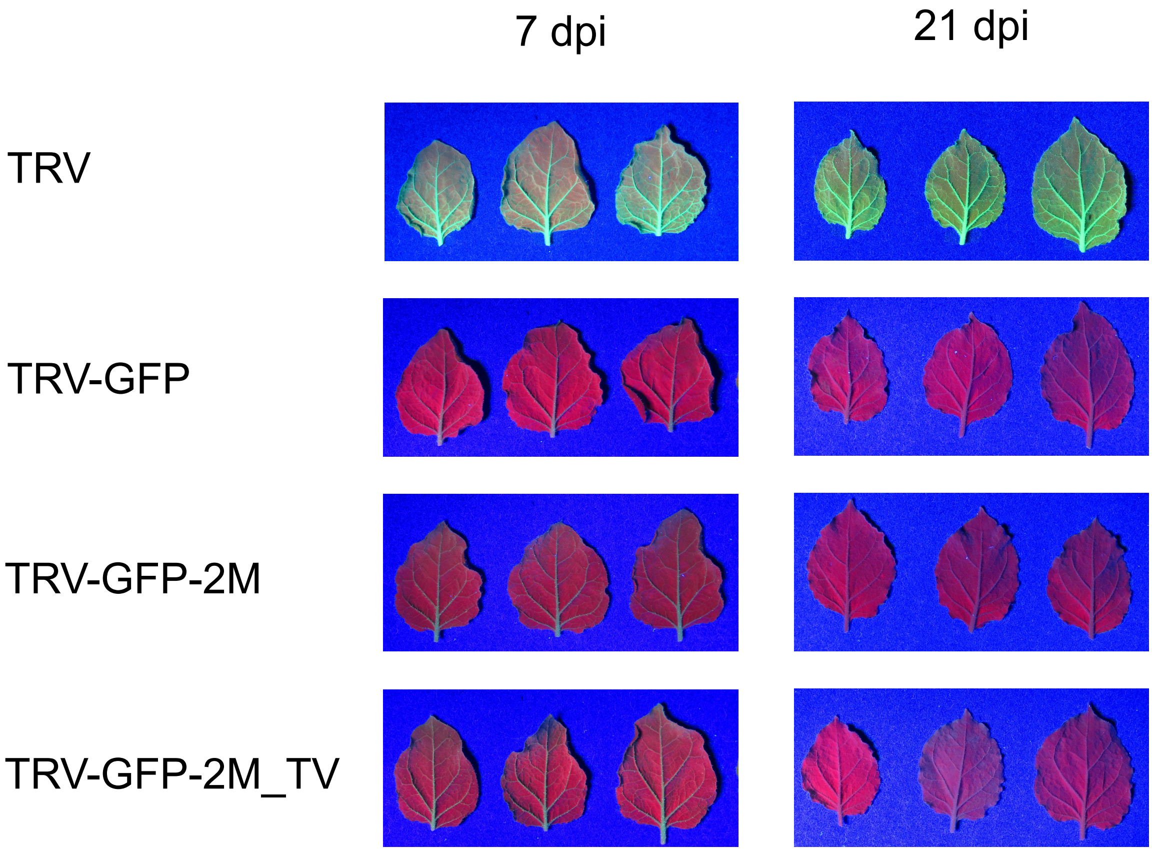

Supplementary Figure S4. Non-perfectly matching small RNAs can induce strong post-transcriptional gene silencing in virus-infected plants. 16c plants were infected with wild type TRV, TRV-GFP, TRV-GFP-2M, and TRV-GFP-2M_TV were photographed under UV light at 7 and 21 dpi.

Supplementary Figure S5

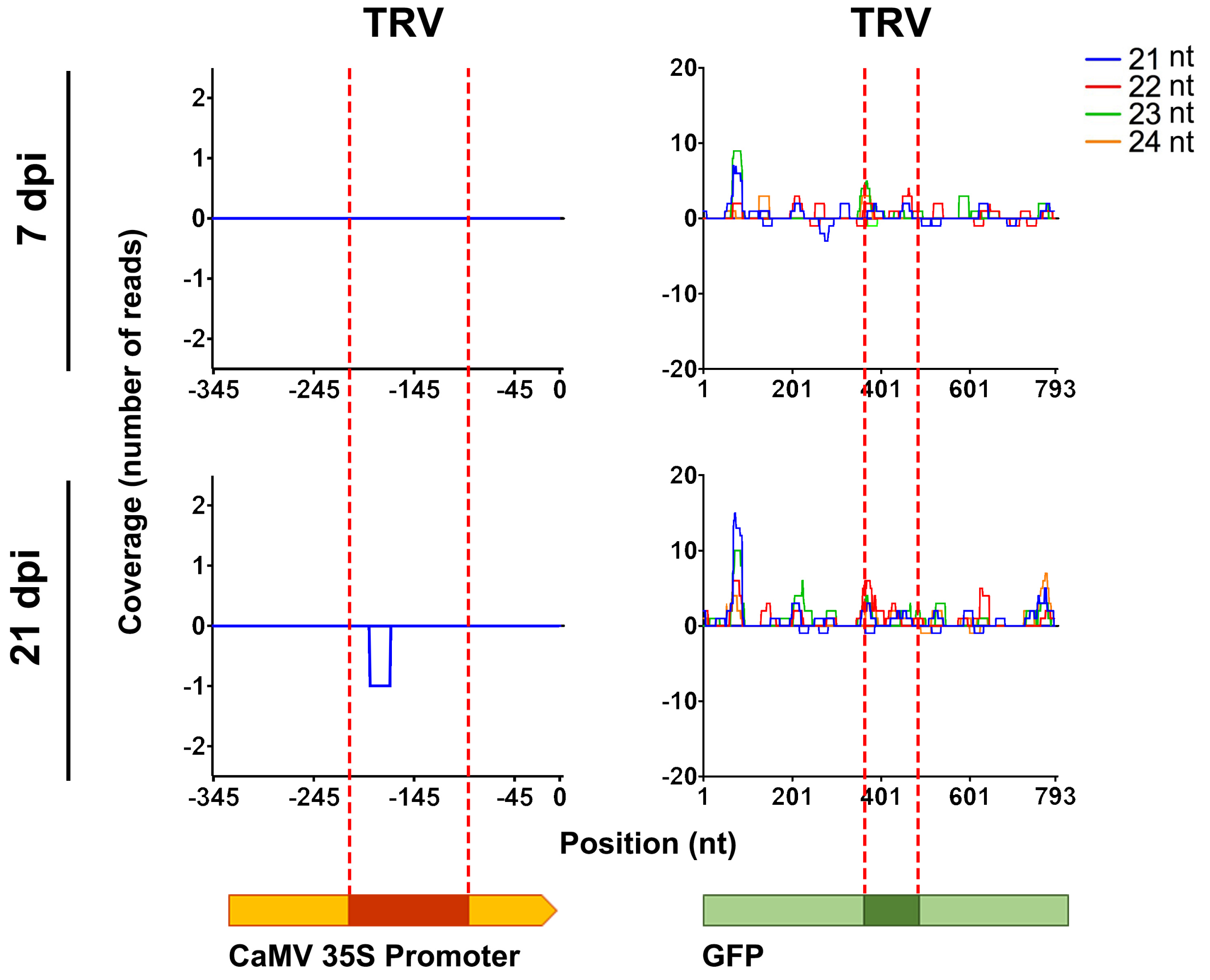

Supplementary Figure S5. **Analysis of small RNAs in TRV-infected *N. benthamiana* 16c plants at 7dpi and 21 dpi.** Small RNA reads were aligned to the 35S promoter and GFP coding sequence. The numbers of sRNAs mapping at each position of the plus strand are shown as positive values, to the minus as negative values, for 21, 22, 23 and 24 nt sRNAs separately. The target sequence is highlighted by dotted lines.

Supplementary Figure S6

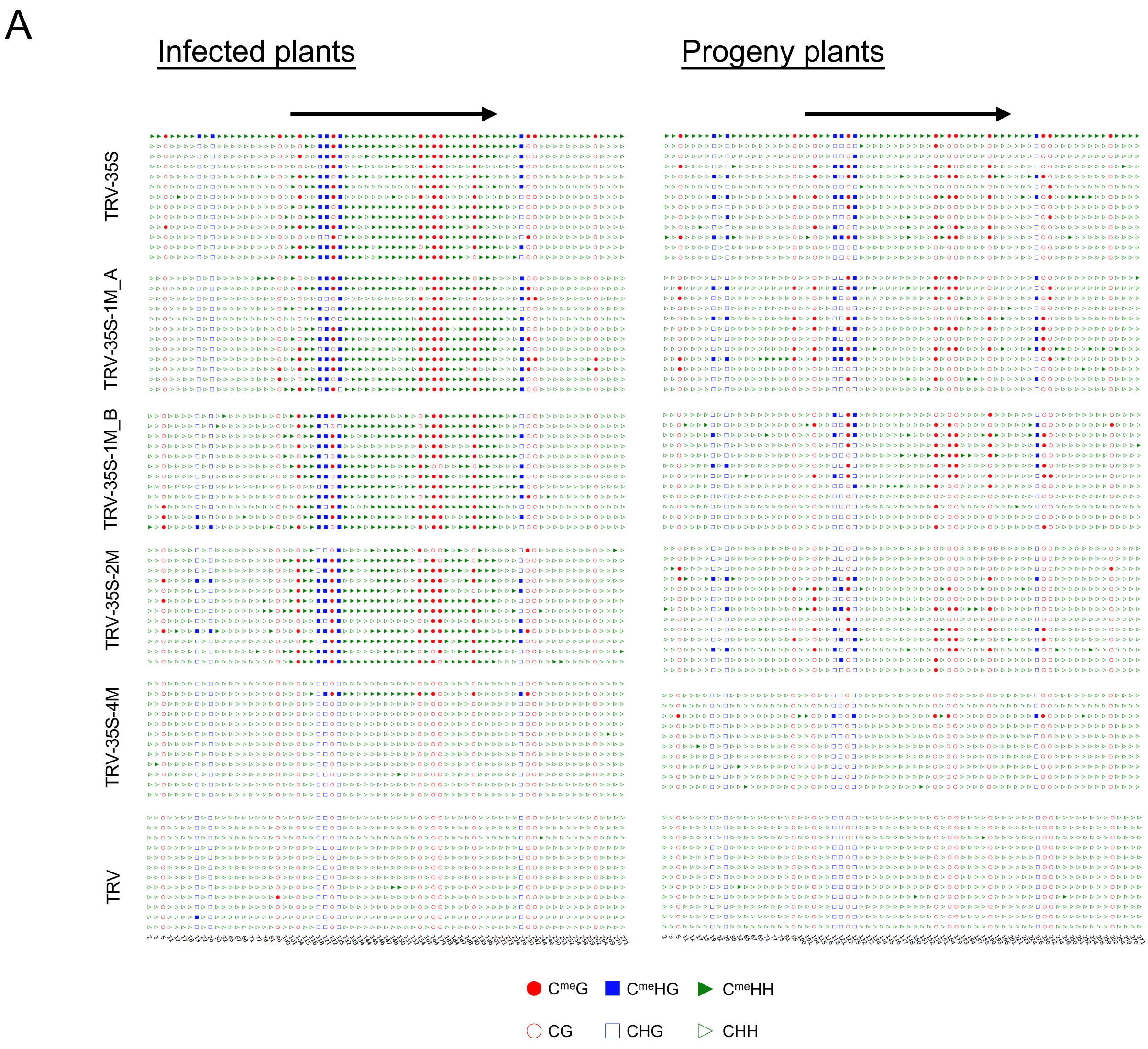

B

		Number of clones	Total C sites	Methylated C sites	% C methylation	95% confidence interval	
						C int-	C int+
Infected plant	TRV-35S	37	1332	885	66.44	63.86	68.93
	TRV-35S-1M_A	25	900	528	58.67	55.42	61.84
	TRV-35S-1M_B	30	1080	654	60.56	57.61	63.43
	TRV-35S-2M	33	1188	734	61.78	58.99	64.51
	TRV-35S-4M	27	972	89	9.16	7.50	11.13
	TRV	35	1260	9	0.71	0.38	1.35
Progeny	TRV-35S	35	1260	209	16.59	14.64	18.74
	TRV-35S-1M_A	30	1080	195	18.06	15.88	20.46
	TRV-35S-1M_B	32	1152	148	12.85	11.04	14.90
	TRV-35S-2M	33	1188	198	16.67	14.66	18.89
	TRV-35S-4M	28	1008	16	1.59	0.98	2.56
	TRV	30	1080	7	0.65	0.31	1.33

		Number of clones	Total CG sites	Methylated CG sites	% CG methylation	95% confidence interval	
						CG int-	CG int+
Infected plant	TRV-35S	37	222	175	78.83	72.99	83.69
	TRV-35S-1M_A	25	150	125	83.33	76.55	88.45
	TRV-35S-1M_B	30	180	145	80.56	74.16	85.67
	TRV-35S-2M	33	198	151	76.26	69.87	81.65
	TRV-35S-4M	27	162	14	8.64	5.22	13.98
	TRV	35	210	3	1.43	0.49	4.12
Progeny	TRV-35S	35	210	94	44.76	38.19	51.52
	TRV-35S-1M_A	30	180	96	53.33	46.05	60.48
	TRV-35S-1M_B	32	192	64	33.33	27.05	40.27
	TRV-35S-2M	33	198	94	47.47	40.63	54.41
	TRV-35S-4M	28	168	4	2.38	0.93	5.96
	TRV	30	180	0	0.00	0.00	2.09

		Number of clones	Total CHG sites	Methylated CHG sites	% CHG methylation	95% confidence interval	
						CHG int-	CHG int+
Infected plant	TRV-35S	37	148	110	74.32	66.73	80.68
	TRV-35S-1M_A	25	100	78	78.00	68.93	85.00
	TRV-35S-1M_B	30	120	101	84.17	76.59	89.62
	TRV-35S-2M	33	132	101	76.52	68.60	82.93
	TRV-35S-4M	27	108	19	17.59	11.56	25.85
	TRV	35	140	1	0.71	0.13	3.94
Progeny	TRV-35S	35	140	58	41.43	33.60	49.71
	TRV-35S-1M_A	30	120	53	44.17	35.60	53.10
	TRV-35S-1M_B	32	128	33	25.78	18.99	33.99
	TRV-35S-2M	33	132	53	40.15	32.18	48.68
	TRV-35S-4M	28	112	4	3.57	1.40	8.83
	TRV	30	120	0	0.00	0.00	3.10

		Number of clones	Total CHH sites	Methylated CHH sites	% CHH methylation	95% confidence interval	
						CHH int-	CHH int+
Infected plant	TRV-35S	37	962	600	62.37	59.27	65.38
	TRV-35S-1M_A	25	650	325	50.00	46.17	53.83
	TRV-35S-1M_B	30	780	408	52.31	48.80	55.79
	TRV-35S-2M	33	858	482	56.18	52.84	59.46
	TRV-35S-4M	27	702	56	7.98	6.19	10.22
	TRV	35	910	5	0.55	0.23	1.28
Progeny	TRV-35S	35	910	57	6.26	4.87	8.03
	TRV-35S-1M_A	30	780	46	5.90	4.45	7.78
	TRV-35S-1M_B	32	832	51	6.13	4.69	7.97
	TRV-35S-2M	33	858	51	5.94	4.55	7.73
	TRV-35S-4M	28	728	8	1.10	0.56	2.15
	TRV	30	780	7	0.90	0.44	1.84

Supplementary Figure S6. Analysis of DNA methylation at the CaMV 35S promoter in recombinant-TRV-infected *N. benthamiana* 16c plants and their progeny. (A) Examination of cytosine methylation by bisulfite sequencing. Raw data is shown from one of the three biological repeats. Up to 12 independent clones of bisulfite-converted DNA were analysed by Cymate from each sample. The position of cytosine residues in the CaMV 35S promoter is indicated at the bottom of the composite figure. CG, CHG and CHH methylation is represented as red circle, blue square and green triangle, respectively. Methylated residues are shown as solid symbols. Black arrow indicates the virus-targeted region of CaMV 35S promoter. **(B)** Raw data from the bisulfite sequencing analysis presented in Figure 4 and Figure 5 including the number of sequenced clones and scored cytosine sites. There are 6 CG sites, 4 CHG sites and 26 CHH sites in the targeted region. The Wilson score was calculated as shown in (1) and (2).

Supplementary Figure S7

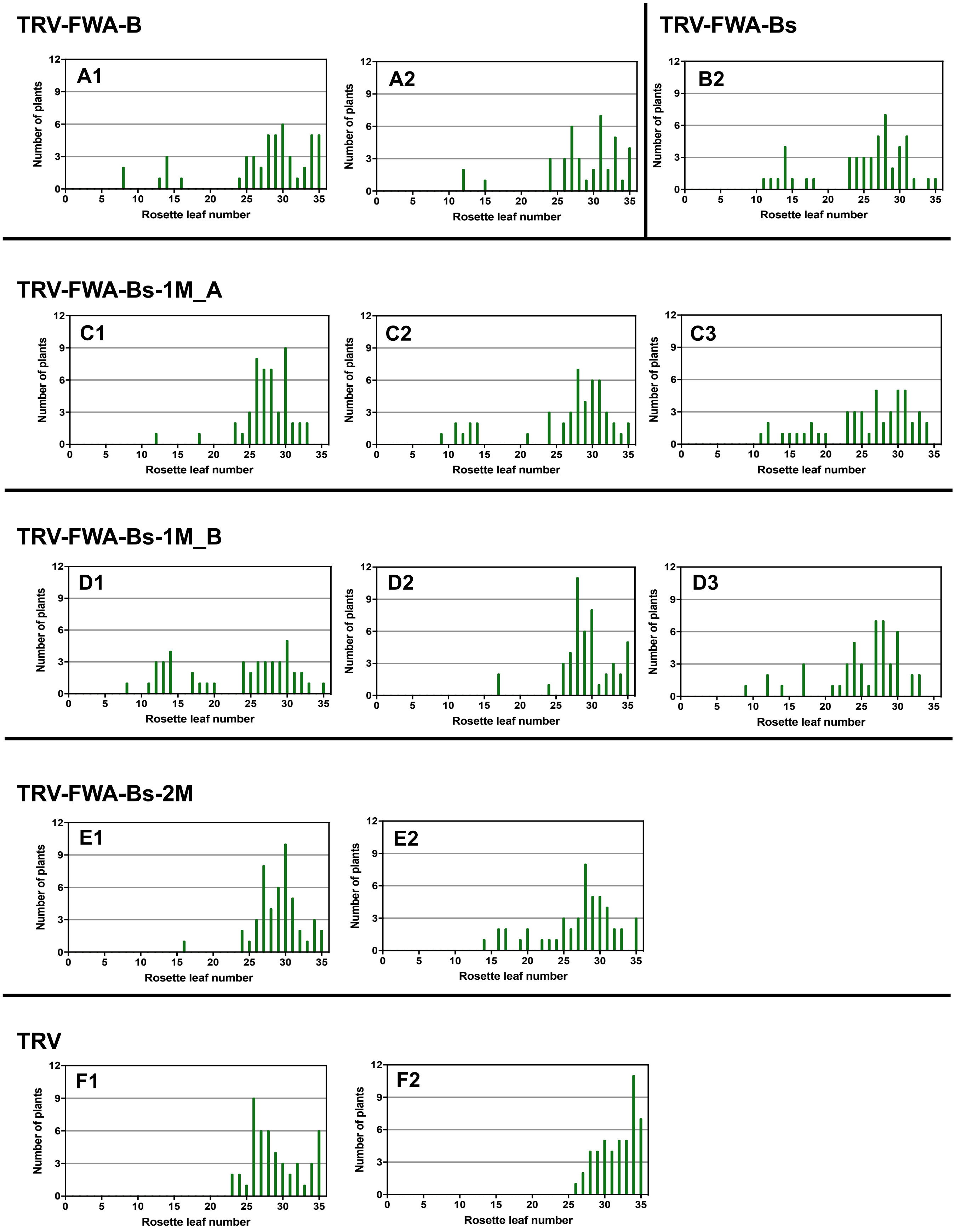

Supplementary Figure S7. Analysis of flowering time (rosette leaf number at the time of bolting) in the progeny of recombinant-TRV-infected *Arabidopsis* plants. Approximately 48 individual plants were analysed from each selected line presented in Figure 7A and B.

Supplementary Figure S8

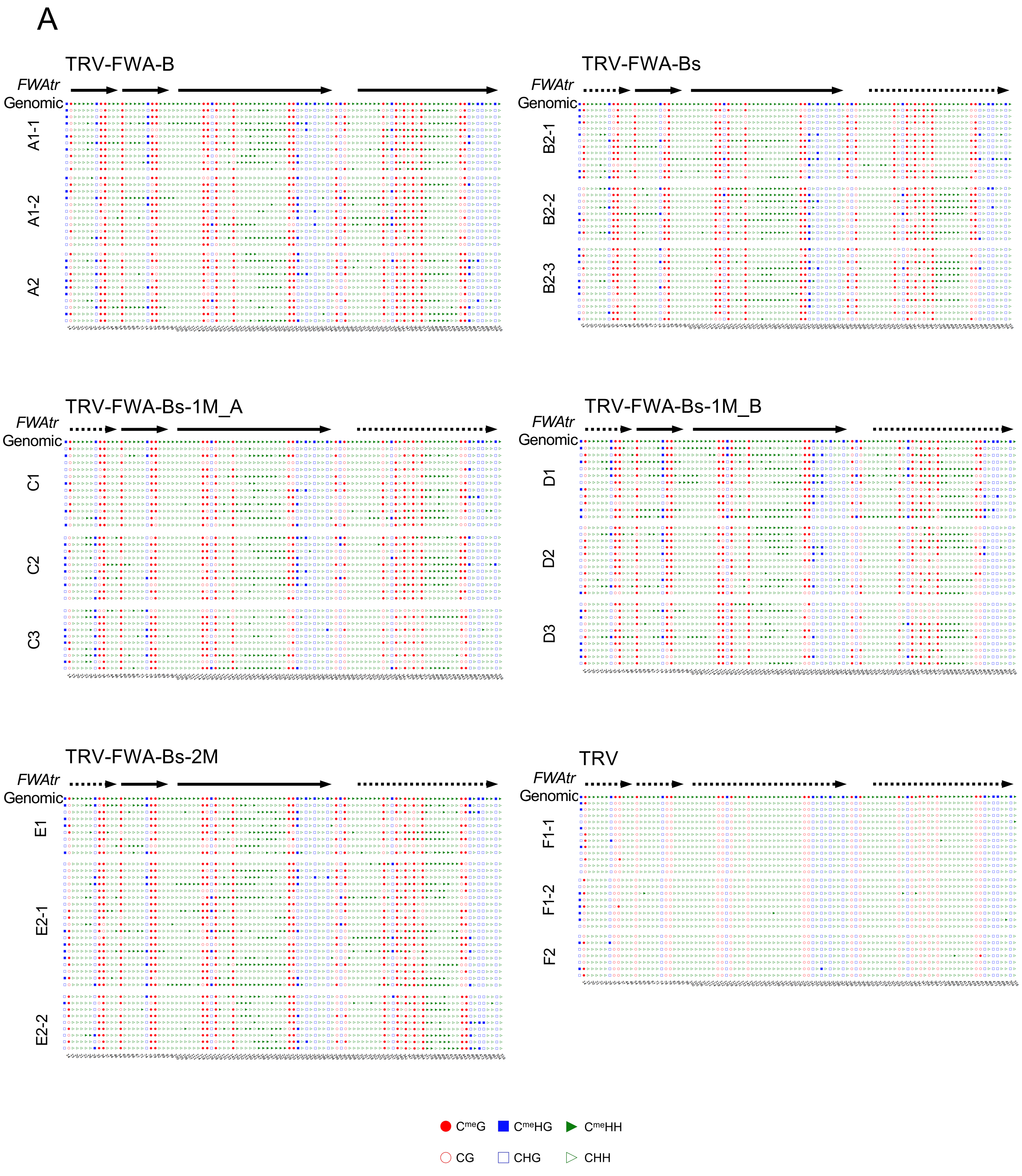

B

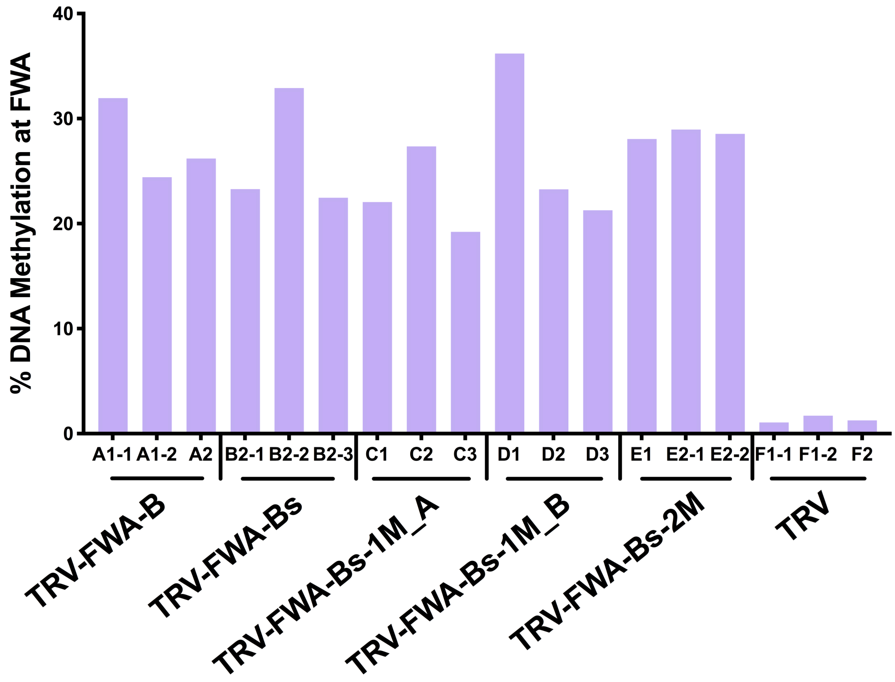

C

	Number of clones	Total C sites	Methylated C sites	% C methylation	95% confidence interval C int-	C int+
TRV-FWA-B	32	3264	894	27.39	25.89	28.95
TRV-FWA-Bs	33	3366	862	25.61	24.16	27.11
TRV-FWA-Bs-1M_A	32	3264	745	22.82	21.42	24.30
TRV-FWA-Bs-1M_B	32	3264	884	27.08	25.59	28.63
TRV-FWA-Bs-2M	36	3672	1052	28.65	27.21	30.13
TRV	27	2754	36	1.31	0.95	1.80

	Number of clones	Total CG sites	Methylated CG sites	% CG methylation	95% confidence interval CG int-	CG int+
TRV-FWA-B	32	672	524	77.98	74.69	80.95
TRV-FWA-Bs	33	693	514	74.17	70.78	77.29
TRV-FWA-Bs-1M_A	32	672	426	63.39	59.68	66.95
TRV-FWA-Bs-1M_B	32	672	449	66.82	63.17	70.27
TRV-FWA-Bs-2M	36	756	590	78.04	74.95	80.85
TRV	27	567	14	2.47	1.48	4.10

	Number of clones	Total CHG sites	Methylated CHG sites	% CHG methylation	95% confidence interval CHG int-	CHG int+
TRV-FWA-B	32	448	69	15.40	12.35	19.04
TRV-FWA-Bs	33	462	66	14.29	11.39	17.77
TRV-FWA-Bs-1M_A	32	438	57	13.01	10.18	16.49
TRV-FWA-Bs-1M_B	32	448	67	14.96	11.95	18.56
TRV-FWA-Bs-2M	36	504	64	12.70	10.07	15.89
TRV	27	378	14	3.70	2.22	6.12

	Number of clones	Total CHH sites	Methylated CHH sites	% CHH methylation	95% confidence interval CHH int-	CHH int+
TRV-FWA-B	32	2144	301	14.04	12.63	15.57
TRV-FWA-Bs	33	2211	282	12.75	11.43	14.21
TRV-FWA-Bs-1M_A	32	2154	262	12.16	10.85	13.61
TRV-FWA-Bs-1M_B	32	2144	368	17.16	15.63	18.82
TRV-FWA-Bs-2M	36	2412	398	16.50	15.07	18.04
TRV	27	1809	8	0.44	0.22	0.87

Supplementary Figure S8. Analysis of DNA methylation at the *FWA* promoter in the progeny of recombinant-TRV-infected Col-0 *fwa-d* plants. (A) Examination of cytosine methylation by bisulfite sequencing. Raw data is generated from three individual plants of each selected line. Up to 12 independent clones of bisulfite-converted DNA were analysed by Cymate from each sample. Labelling as in Supplementary Figure 6. Black arrows indicate the position of FWA tandem repeats. Solid black arrows indicate the virus-targeted region of FWA promoter. **(B)** Analysis of the percentage of total methylated cytosine at the *FWA* promoter in each individual plant. **(C)** Raw data from the bisulfite sequencing analysis presented in Figure 7 including the number of sequenced clones and scored cytosine sites. There are 21 CG sites, 14 CHG sites and 67 CHH sites in the amplified region.

Supplementary Figure S9

```
2nd-LR                     1    TTATCCCATTCAACATTCATACGAGCACCGCTTTACGGTTTTTGCTTTTC
1st-LR                     1    TTATCCCATTCAACATTCATACGAGCGCCGCTCTAGGGTTTTTGCTTTTC
1st-LR in FWA-Bs-1M_A      1    TTATCCCACTCAACATTCATACGAGCGCTGCTCTAGGGTTTTTGCTTTCC
1st-LR in FWA-Bs-1M_B      1    TTATCCCATTCAACATTCGTACGAGCGCCGCTCTAGGGCTTTTGCTTTTC
1st-LR in FWA-Bs-2M        1    TTATCCCACTCAACATTCGTACGAGCGCTGCTCTAGGGCTTTTGCTTTCC

2nd-LR                     51   GACATTGGTCGAAGTGCTATTTGGTTGTTTAAGGTTGCTTTTAGCACACA
1st-LR                     51   GCCATTGGTCCAAGTGCTATTTGGTTGTTTAAGGTTGCTTTTAGCACACA
1st-LR in FWA-Bs-1M_A      51   GCCATTGGTCCAAGTGCTGTTTGGTTGTTTAAGGTTGCCTTTAGCACACA
1st-LR in FWA-Bs-1M_B      51   GCCATTGGCCCAAGTGCTATTTGGTTGTCTAAGGTTGCTTTTAGCACATA
1st-LR in FWA-Bs-2M        51   GCCATTGGCCCAAGTGCTGTTTGGTTGTCTAAGGTTGCCTTTAGCACATA

2nd-LR                     101  ACTTTAATATTATTTTTATGTT-TTCTTCTTACGATTTATCGATTTGTAG
1st-LR                     101  ACTTTAATATTATTTTTATGTTTTTCTTCTTACGATTTATCGATTTGTGG
1st-LR in FWA-Bs-1M_A      101  ACTTTAATGTTATTTTTATGTTTTTCTTTTTACGATTTATCGATTTGTTG
1st-LR in FWA-Bs-1M_B      101  ACTTTAATATTATTTTTACGTTTTTCTTCTTACGATTTGTCGATTTGTGG
1st-LR in FWA-Bs-2M        101  ACTTTAATGTTATTTTTACGTTTTTCTTTTTACGATTTGTCGATTTGTTG

2nd-LR                     150  GATACTGACAATCAGATTTTTGTTGTTTTTTTCAGCCAAAAATCAGAT
1st-LR                     151  GATACTGACAATCAGATTATTGTTGTTTTTTCCAGCCAAATATCAGAT
1st-LR in FWA-Bs-1M_A      151  GATACTGACAATCAGATTGTTGTTGTTTTTTCCAGCCAGATATCAGAT
1st-LR in FWA-Bs-1M_B      151  GATACTGATAATCAGATTATTGTTGTTTCTTCCAGCCAAATATCAGAT
1st-LR in FWA-Bs-2M        151  GATACTGATAATCAGATTGTTGTTGTTTCTTCCAGCCAGATATCAGAT
```

Supplementary Figure S9. Sequence alignment of FWA long repeats and derivatives.

Supplementary Figure S10

A

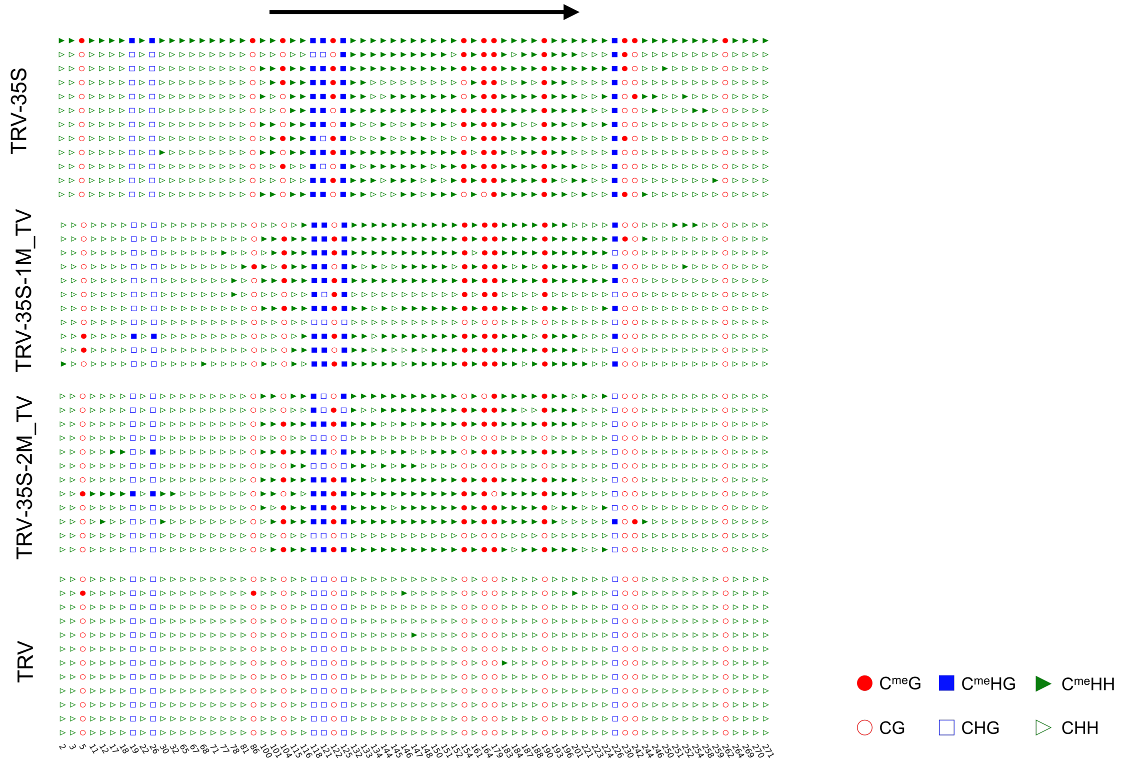

B

	Number of clones	Total C sites	Methylated C sites	% C methylation	95% confidence interval C int-	C int+
TRV-35S	35	1260	896	71.11	68.55	73.55
TRV-35S-1M_AT	34	1224	826	67.48	64.81	70.05
TRV-35S-2M_AT	36	1296	797	61.50	58.52	64.11
TRV	35	1260	8	0.63	0.3221	1.248

	Number of clones	Total CG sites	Methylated CG sites	% CG methylation	95% confidence interval CG int-	CG int+
TRV-35S	35	210	178	84.76	79.28	88.99
TRV-35S-1M_AT	34	204	162	79.41	73.34	84.39
TRV-35S-2M_AT	36	216	126	58.33	51.67	64.71
TRV	35	210	0	0.00	0	1.796

	Number of clones	Total CHG sites	Methylated CHG sites	% CHG methylation	95% confidence interval CHG int-	CHG int+
TRV-35S	35	140	123	87.86	81.41	92.28
TRV-35S-1M_AT	34	136	106	77.94	70.26	84.09
TRV-35S-2M_AT	36	144	80	55.56	47.4	63.42
TRV	35	140	0	0.00	0	2.671

	Number of clones	Total CHH sites	Methylated CHH sites	% CHG methylation	95% confidence interval CHH int-	CHH int+
TRV-35S	35	910	595	65.38	62.23	68.41
TRV-35S-1M_AT	34	884	558	63.12	59.89	66.24
TRV-35S-2M_AT	36	936	591	63.14	60	66.17
TRV	35	910	8	0.88	0.4461	1.725

Supplementary Figure S10. Analysis of DNA methylation at the CaMV 35S promoter in recombinant-TRV-infected *N. benthamiana* 16c plants. (A) Examination of cytosine methylation by bisulfite sequencing. Raw data is shown from one of the three biological repeats. Up to 12 independent clones of bisulfite-converted DNA were analysed by Cymate from each sample. The position of cytosine residues in the CaMV 35S promoter is indicated at the bottom of the composite figure. CG, CHG and CHH methylation is represented as red circle, blue square and green triangle, respectively. Methylated residues are shown as solid symbols. Black arrow indicates the virus-targeted region of CaMV 35S promoter. **(B)** Raw data from the bisulfite sequencing analysis presented in Figure 8E and F including the number of sequenced clones and scored cytosine sites. There are 6 CG sites, 4 CHG sites and 26 CHH sites in the targeted region.

Supplementary Figure S11

A

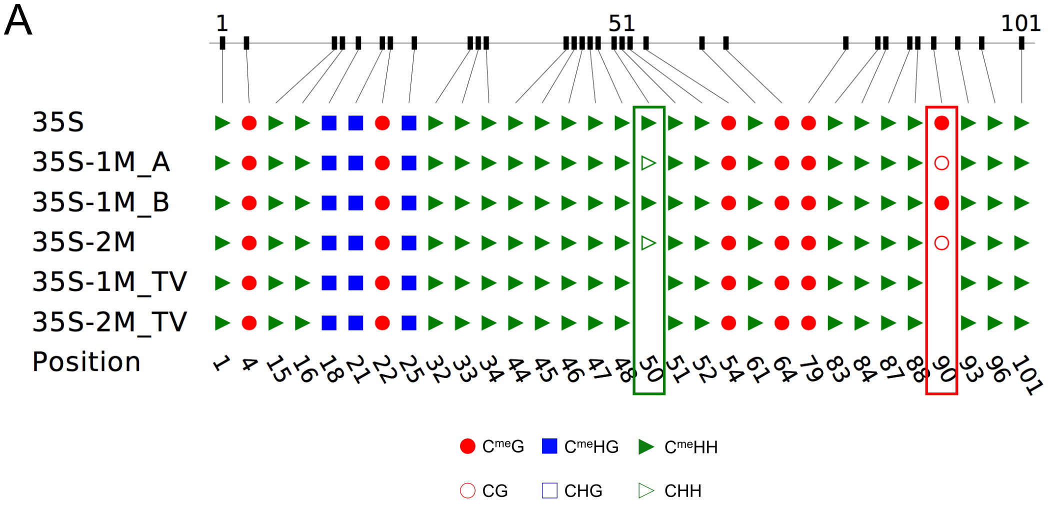

B

	Number of clones	Position_50 CHH			Position_90 CG		
		Methylated sites	% methylation	% methylation (global)	Methylated sites	% methylation	% methylation (global)
TRV-35S	37	26	70	62	30	81	79
TRV-35S-1M_A	25	11	44	50	21	84	83
TRV-35S-1M_B	30	12	40	52	29	97	81
TRV-35S-2M	33	17	52	56	26	79	76
TRV-35S-1M_TV	34	24	71	63	30	88	79
TRV-35S-2M_TV	36	26	72	63	24	67	58

Supplementary Figure S11. Analysis of the effect of direct and indirect pairing of mismatched nucleotides on cytosine methylation at the CaMV 35S promoter in recombinant-TRV-infected *N. benthamiana* 16c plants. (A) Schematic diagram of the position of cytosine residues in the CaMV 35S promoter. CG, CHG and CHH methylation is represented as red circle, blue square and green triangle, respectively. Cytosine residues directly affected by SNS in recombinant TRV/small RNA are shown as empty symbols, and their positions are highlighted in boxes. **(B)** Analysis of bisulfite sequencing data presented in Figure 4 and 8.

Supplementary Table S1. Analysis of sRNA libraries. Related to Figure 3 and Figure S2.

sRNA library	Total reads	Filtered reads (21-24 nt)	Total reads for			% 120 nt/CP
			TRV-RNA2	TRV CP	TRV 120 nt insert	
TRV-35S 7dpi	11,647,785	5,667,519	722,409	230,158	10,292	4.47
TRV-35S 21dpi	11,506,628	6,233,872	610,988	221,652	8,587	3.87
TRV-35S-2M 7dpi	12,521,906	6,007,339	681,260	209,363	12,955	6.19
TRV-35S-2M 21dpi	10,670,771	3,593,089	459,278	147,162	8,997	6.11
TRV-GFP 7dpi	10,535,377	5,922,424	746,512	237,707	20,932	8.81
TRV-GFP 21dpi	10,967,090	4,073,630	409,917	140,555	14,244	10.13
TRV-GFP-2M 7dpi	11,557,069	6,458,851	520,451	159,420	14,970	9.39
TRV-GFP-2M 21dpi	12,457,417	5,635,874	500,807	166,749	14,760	8.85

Supplementary Table S2. Oligonucleotides used in this study.

Oligonucleotide name	Sequence	Description
35S-1M_A forward	CATCGTTGAGGATGCCTCTGCCGACAGTGATCCCAAAGATGGACCCCCATCCACGAGGAGCATCGTGGAGAAAGAAGACGTTCCAACCATGTCTTCAAAGCAAGTGGATCGATGTGATAT	VIGS
35S-1M_A reverse	ATATCACATCGATCCACTTGCTTTGAAGACATGGTTGGAACGTCTTCTTTCTCCACGATGCTCCTCGTGGATGGGGGTCCATCTTTGGGATCACTGTCGGCAGAGGCATCCTCAACGATG	VIGS
35S-1M_B forward	CATCGTTGAAGATGCCTCTACCGACAGTGGTCCCAAAGACGGACCCCCACCCACGAGGAACATCGTGGAAAAAGAAGACATTCCAACCACGTCTTCAAAACAAGTGGATTGATGTGATAC	VIGS
35S-1M_B reverse	GTATCACATCAATCCACTTGTTTTGAAGACGTGGTTGGAATGTCTTCTTTTTCCACGATGTTCCTCGTGGGTGGGGGTCCGTCTTTGGGACCACTGTCGGTAGAGGCATCTTCAACGATG	VIGS
35S-2M forward	CATCGTTGAGGATGCCTCTACCGACAGTGATCCCAAAGACGGACCCCCATCCACGAGGAACATCGTGGAGAAAGAAGACATTCCAACCATGTCTTCAAAACAAGTGGATCGATGTGATAC	VIGS
35S-2M reverse	GTATCACATCGATCCACTTGTTTTGAAGACATGGTTGGAATGTCTTCTTTCTCCACGATGTTCCTCGTGGATGGGGGTCCGTCTTTGGGATCACTGTCGGTAGAGGCATCCTCAACGATG	VIGS
35S-4M forward	CATCATTGAGGATGTCTCTACCGATAGTGATCCCGAAGACGGACTCCCATCCACAAGGAACATCATGGAGAAAGGAGACATTCCGACCATGTCTCCAAAACAAGCGGATCGATGCGATAC	VIGS
35S-4M reverse	GTATCGCATCGATCCGCTTGTTTTGGAGACATGGTCGGAATGTCTCCTTTCTCCATGATGTTCCTTGTGGATGGGAGTCCGTCTTCGGGATCACTATCGGTAGAGACATCCTCAATGATG	VIGS
35S-1M_TV forward	CATCGTTGATGATGCCTCTGCCGACAGTGCTCCCAAAGATGGACCCCCAGCCACGAGGAGCATCGTGGATAAAGAAGACGTTCCAACCAGGTCTTCAAAGCAAGTGGATAGATGTGATAT	VIGS
35S-1M_TV reverse	ATATCACATCTATCCACTTGCTTTGAAGACCTGGTTGGAACGTCTTCTTTATCCACGATGCTCCTCGTGGCTGGGGGTCCATCTTTGGGAGCACTGTCGGCAGAGGCATCATCAACGATG	VIGS
35S-2M_TV forward	CATCGTTGATGATGCCTCTCCCGACAGTGCTCCCAAAGAAGGACCCCCAGCCACGAGGACCATCGTGGATAAAGAAGACCTTCCAACCAGGTCTTCAAACCAAGTGGATAGATGTGATAA	VIGS
35S-2M_TV reverse	TTATCACATCTATCCACTTGGTTTGAAGACCTGGTTGGAAGGTCTTCTTTATCCACGATGGTCCTCGTGGCTGGGGGTCCTTCTTTGGGAGCACTGTCGGGAGAGGCATCATCAACGATG	VIGS
FWA-Bs-1M_A top	CGAAGCCCACACATCTTTCCGTCGAGAATTTCATATATACCTTATCCCACTCAACATTCATACGAGCGCTGCTCTAGGGTTTTTGCTTTCCGCCATTGGT	VIGS
FWA-Bs-1M_A middle	AAAGAAAAACATAAAAATAACATTAAAGTTGTGTGCTAAAGGCAACCTTAAACAACCAAACAGCACTTGGACCAATGGCGGAAAGCAAAAACCCTAGAGC	VIGS
FWA-Bs-1M_A bottom	CAACTTTAATGTTATTTTTATGTTTTTCTTTTTACGATTTATCGATTTGTTGGATACTGACAATCAGATTGTTGTTGTTTTTTCCAGCCAGATATCAGAT	VIGS
FWA-Bs-1M_B top	CGAAGCCCATACATCTTTCTGTCGAGAATCTCATATATATCTTATCCCATTCAACATTCGTACGAGCGCCGCTCTAGGGCTTTTGCTTTTCGCCATTGGC	VIGS
FWA-Bs-1M_B middle	GAAGAAAAACGTAAAAATAATATTAAAGTTATGTGCTAAAAGCAACCTTAGACAACCAAATAGCACTTGGGCCAATGGCGAAAAGCAAAAGCCCTAGAGC	VIGS
FWA-Bs-1M_B bottom	TAACTTTAATATTATTTTTACGTTTTTCTTCTTACGATTTGTCGATTTGTGGGATACTGATAATCAGATTATTGTTGTTTCTTCCAGCCAAATATCAGAT	VIGS
FWA-Bs-2M top	CGAAGCCCACACATCTTTCTGTCGAGAATTTCATATATATCTTATCCCACTCAACATTCGTACGAGCGCTGCTCTAGGGCTTTTGCTTTCCGCCATTGGC	VIGS
FWA-Bs-2M middle	AAAGAAAAACGTAAAAATAACATTAAAGTTATGTGCTAAAGGCAACCTTAGACAACCAAACAGCACTTGGGCCAATGGCGGAAAGCAAAAGCCCTAGAGC	VIGS
FWA-Bs-2M bottom	TAACTTTAATGTTATTTTTACGTTTTTCTTTTTACGATTTGTCGATTTGTTGGATACTGATAATCAGATTGTTGTTGTTTCTTCCAGCCAGATATCAGAT	VIGS
FWA-Bs forward	CGAAGCCCATACATCTTTCCG	VIGS
FWA-Bs reverse	ATCTGATATTTGGCTGGAAAA	VIGS
FWA-Bs-1M_A forward	CGAAGCCCACACATCTTTCCG	VIGS
FWA-Bs-1M_A reverse	ATCTGATATCTGGCTGGAAAA	VIGS
FWA-Bs-1M_B forward	CGAAGCCCATACATCTTTCTG	VIGS
FWA-Bs-1M_B reverse	ATCTGATATTTGGCTGGAAGA	VIGS
FWA-Bs-2M forward	CGAAGCCCACACATCTTTCTG	VIGS
FWA-Bs-2M reverse	ATCTGATATCTGGCTGGAAGA	VIGS

Oligonucleotide name	Sequence	Description
GFP +364 forward	AAGGACGACGGGAACTACAAG	VIGS
GFP +483 reverse	CTTGTGGCCGAGGATGTTTC	VIGS
GFP-2M forward	AAGGACGACAGGAACTACAGGACACGTGCCGAAGTCAAGCTTGAGGGAGGC ACCCTCGTTAACAGGATCAAGCTTAAGGAAATCGATTTTAAGGAGGACAGAA ACATCCCCGGCCACAAA	VIGS
GFP-2M reverse	TTTGTGGCCGGGGATGTTTCTGTCCTCCTTAAAATCGATTTCCTTAAGCTTGA TCCTGTTAACGAGGGTGCCTCCCTCAAGCTTGACTTCGGCACGTGTCCTGTA GTTCCTGTCGTCCTT	VIGS
GFP-2M_TV forward	AAGGACGACCGGAACTACATGACACGTGCAGAAGTCAAGATTGAGGGAGTC ACCCTCGTGAACAGGATCCAGCTTAAGGCAATCGATTTGAAGGAGGACCGAA ACATCCACGGCCACAAC	VIGS
GFP-2M_TV reverse	GTTGTGGCCGTGGATGTTTCGGTCCTCCTTCAAATCGATTGCCTTAAGCTGG ATCCTGTTCACGAGGGTGACTCCCTCAATCTTGACTTCTGCACGTGTCATGTA GTTCCGGTCGTCCTT	VIGS
35S_bisulfite forward	TGAGATTTTTTAATAAAGGGTAATTTYGGGAAATT	Bisulfite sequencing PCR
35S_bisulfite reverse	TCCTCTCCAAATAAAATAAACTTCCTTATATAAAAA	Bisulfite sequencing PCR
FWA_bisulfite forward	ATTAAAGAGTTATGGGYYGAAGTTTAT	Bisulfite sequencing PCR
FWA_bisulfite reverse	CRRRAACCAAAATCATTCTCTAAACA	Bisulfite sequencing PCR
M13 forward	CGCCAGGGTTTTCCCAGTCACGAC	Colony PCR
M13 reverse	AGCGGATAACAATTTCACACAGGA	Colony PCR
Nb-act forward	GAAGATACTCACAGAAAGAGG	qRT-PCR
Nb-act reverse	GGAGCTAATGCAGTAATTTCC	qRT-PCR
AtEF1a forward	CACCACTGGAGGTTTTGAGG	qRT-PCR
AtEF1a reverse	TGGAGTATTTGGGGGTGGT	qRT-PCR
TRV-CP-713 forward	TGGGTTACTAGCGGCACTGA	qRT-PCR
TRV-CP-856 reverse	GCTCGTCTCTTGAACGCTGA	qRT-PCR
mGFP5 +148 forward	ACTGGAAAACTACCTGTTCC	qRT-PCR
mGFP5 +344 reverse	TCAAACTTGACTTCAGCACG	qRT-PCR
Insert forward	AATCTCAGACTGTTCCGCTTC	qRT-PCR
Insert reverse	GATGGACAACCCGTTCACCAC	qRT-PCR

References

1. Henderson, I.R., Deleris, A., Wong, W., Zhong, X., Chin, H.G., Horwitz, G.A., Kelly, K.A., Pradhan, S. and Jacobsen, S.E. (2010) The de novo cytosine methyltransferase DRM2 requires intact UBA domains and a catalytically mutated paralog DRM3 during RNA-directed DNA methylation in Arabidopsis thaliana. *PLoS Genet*, **6**, e1001182.

2. Jullien, P.E., Susaki, D., Yelagandula, R., Higashiyama, T. and Berger, F. (2012) DNA methylation dynamics during sexual reproduction in Arabidopsis thaliana. *Curr Biol*, **22**, 1825-1830.
